# Heterogeneous income gradients in early motherhood in Mexico: a population-based multi-method study, 2000–2020

**DOI:** 10.1186/s12939-026-02908-w

**Published:** 2026-06-25

**Authors:** Edson Serván-Mori, Alejandro Zamudio-Sosa, Lucio Esposito, Gabriela Sandoval-Moreno, H. Xavier Jara, Daniella Medeiros Cavalcanti, Ana L. Moncayo, Davide Rasella, Carlos Chivardi

**Affiliations:** 1 Center for Health Systems Research, National Institute of Public Health, Cuernavaca, Mexico; 2 Universidad Icesi, Cali, Colombia; 3 Centro de Investigación para la Salud en América Latina, Pontificia Universidad Católica del Ecuador, Quito, Ecuador; 4 London School of Economics and Political Science, London, UK; 5 The Institute of Collective Health, Federal University of Bahia, Bahia, Brazil; 6 The Barcelona Institute of Global Health (ISGlobal), Barcelona, Spain; 7 The Catalan Institution for Research and Advanced Studies (ICREA), Barcelona, Spain; 8 Centre for Health Economics, University of York, York, YO10 5DD UK

**Keywords:** Early motherhood, Household income, Reproductive health inequities, Social protection fragmentation, Social determinants, Mexico

## Abstract

**Background:**

Early motherhood remains a major public health and equity challenge in low- and middle-income countries, especially in Latin America. While poverty, education, gender norms, and barriers to sexual and reproductive health are well-established determinants, how household income translates into (or fails to translate into) delayed childbearing remains insufficiently understood—particularly in highly unequal settings marked by informality and fragmented social protection.

**Objective:**

To examine the association between household income and early motherhood in Mexico, and to disentangle direct and indirect socioeconomic pathways linking income to early childbearing.

**Methods:**

We conducted a population-based, multi-method analysis using harmonised municipal microdata from the 2000, 2010, and 2020 Mexican Population and Housing Censuses (*n* = 3.05 million women aged 12–24 years, representing 30.6 million individuals). Total household income was imputed using Quantile Random Forests trained on nationally representative income–expenditure surveys. Our analytical strategy combined: (1) machine learning models to identify key correlates and non-linear individual-level patterns of early motherhood; (2) Bayesian spatial models (INLA) to quantify subnational clustering and contextual heterogeneity; and (3) dynamic structural equation modelling to assess direct and lagged relationships among income, schooling-related indicators, union formation, and early motherhood outcomes.

**Results:**

Early motherhood prevalence declined modestly from 21.46% (2000) to 19.31% (2020) and remained concentrated in socioeconomically disadvantaged households. In individual-level models, marital status, age, household structure, and dependency ratio were the dominant correlates; income ranked eighth in feature importance. Partial dependence plots indicated a non-linear inverse L-shaped association: predicted risk increased up to approximately Int$ 1,760 (2018 PPP) per adult equivalent, then plateaued and declined slightly. Parity-specific models showed that income’s predictive relevance weakened with higher parity, with U-shaped patterns at the lowest income levels for second and ≥third births. Municipal analyses revealed persistent spatial clustering after adjustment for key predictors, and residual “place” effects consistent with unmeasured contextual constraints shaping early motherhood. In dynamic models, the direct income–early motherhood path was not statistically significant. Overall patterns were consistent with predominantly indirect linkages operating through schooling- and union-related indicators and reinforcing disadvantage dynamics at the municipal level.

**Conclusions:**

In Mexico, early motherhood remains strongly shaped by intersecting social and territorial inequalities. Income gradients are modest and appear to operate mainly through schooling lag and union-formation pathways rather than as an isolated driver. Equity-oriented strategies are likely to be most effective when they integrate school retention, prevention of early unions, and youth-friendly sexual and reproductive health services, prioritising municipalities with persistently elevated risk.

**Supplementary Information:**

The online version contains supplementary material available at https://doi.org/10.1186/s12939-026-02908-w.

## Research in context box

What is already known about the topic?

What does this study add to the literature?

What are the policy implications?

• Early motherhood is a persistent equity challenge in many LMICs, including Mexico, and is strongly patterned by socioeconomic disadvantage and unequal opportunity structures.

• Established correlates include school discontinuity, early unions, constrained access to youth-friendly sexual and reproductive health services, and gendered norms.

• Prior evidence on the relationship between household economic resources and early motherhood is mixed and often relies on cross-sectional or aggregate indicators that can obscure non-linear gradients and subnational heterogeneity.

• Using harmonised long-form Mexican census microdata (2000–2020) for 3.05 million women aged 12–24 years, we examine early motherhood through a complementary multi-method framework spanning individual, household, and municipal levels.

• We identify a non-linear inverse L-shaped income gradient: predicted risk rises up to a modest threshold (~ Int-$PPP 1,760 per adult equivalent), then stabilises and declines only slightly; income is less predictive than marital/union status (married/common-law), age, household structure, and demographic dependency.

• Spatial analyses identify geographic clustering of high-risk municipalities, and dynamic models suggest income operates mainly through indirect pathways via schooling and marital/union-related indicators, consistent with reinforcing feedback loops between educational disadvantage and marital/union composition.

• Income gains alone are unlikely to produce large reductions in early motherhood unless they translate into sustained school retention and reduced exposure to early unions, especially in disadvantaged households and high-risk territories.

• Integrated strategies should combine: (i) keeping girls in secondary/upper-secondary education, (ii) preventing early unions and expanding life-course opportunities, and (iii) accessible, youth-friendly SRH services (including effective contraception).

• Because risk is geographically clustered and shaped by interacting social mechanisms, interventions should be territorially targeted and coordinated across education, health, and social protection systems—strengthening coherent service delivery in municipalities with persistently elevated risk.

**Supplementary Information:**

The online version contains supplementary material available at https://doi.org/10.1186/s12939-026-02908-w.

## Background

Despite considerable declines in global fertility rates, adolescent motherhood remains a common life-course event in many low- and middle-income countries (LMICs) [1–4]. Each year, approximately 12 million girls aged 15–19 give birth globally, with nearly 800,000 of these births occurring before the age of 15 [5]. The burden is particularly high in Latin America and the Caribbean (LAC), where the adolescent fertility rate stands at 53 births per 1,000 girls aged 15–19, making it the second-highest in the world, surpassed only by sub-Saharan Africa [6, 7]. In Mexico, adolescent fertility has declined only modestly over the past two decades, with the adolescent birth rate averaging around 70 births per 1,000 girls aged 15–19, despite improvements in education and poverty reduction [8–10].

Adolescent motherhood is associated with adverse health, educational, and economic outcomes for young women and their children, including higher risks of maternal health problems, neonatal death, school dropout, and long-term economic difficulties [9, 11–14]. It perpetuates intergenerational cycles of disadvantage that hinder progress toward multiple Sustainable Development Goals (SDGs), particularly those related to health, education, gender equality, and reducing inequality [9, 12, 15–18].

Research consistently highlights poverty, limited education, gender norms, and restricted access to reproductive health services as key factors influencing adolescent motherhood [19–24]. However, the income–early childbearing relationship is not uniform across settings, and the pathways through which income influences the timing of a first birth remain insufficiently characterised in contexts marked by persistent inequality [9, 17, 23–29]. Economic and social theories suggest multiple interacting pathways: material pathways (resources to support schooling, contraception, and healthcare), opportunity-cost pathways (higher expected returns to education and work delaying childbearing), and psychosocial pathways (aspirations, stress, and perceived life chances) [30–36]. In highly unequal settings, these mechanisms may be modified or constrained by structural conditions—such as labour-market informality, segmented welfare institutions, and gendered norms—that shape opportunities and access to services, potentially weakening the protective effect of rising incomes and contributing to heterogeneity across social groups and territories [30–36].

Many studies rely on cross-sectional surveys or aggregate indicators, which can obscure non-linearities and subnational heterogeneity in income-related patterns of adolescent childbearing [6, 9, 37–41]. Few analyses jointly evaluate household economic resources, territorial clustering, and potential mediating pathways linking income and early childbearing within a single empirical framework [6, 9, 37–40]. Evidence is also limited on income-related patterns when early motherhood is examined across the transition from adolescence into early young adulthood (e.g., 12–24 years), which census microdata can capture consistently over time [42]. In this study, we therefore operationalise early motherhood over ages 12–24 to capture reproductive transitions observable in harmonised long-form census microdata, while maintaining the conventional 15–19 metric for contextual comparison. This operational choice is also consistent with contemporary developmental and social-role evidence, according to which biological maturation, neurocognitive development, and key transitions—including schooling, labour market entry, union formation, and parenthood—commonly extend into the early twenties [42]. This operational choice should not be understood as implying that all processes within ages 12–24 are identical; rather, it defines the age range over which early motherhood is examined in the available census data. We use early motherhood for the 12–24 operational measure and reserve adolescent motherhood for the conventional 15–19 benchmark.

Mexico provides a relevant case because it combines large regional inequalities, a sizeable informal labour market, and a fragmented social protection system [43–46]. While total fertility has fallen, adolescent fertility has remained comparatively high and socially patterned, with persistent concentration among rural, indigenous, and low-income populations [8–10, 41, 47]. This combination makes Mexico well-suited to examine whether associations between income and early childbearing vary across the income distribution and across territories with different opportunity structures and service environments.

In this study, we use harmonised census microdata (2000–2020) to examine heterogeneous associations between household income and early motherhood among adolescents and young women in Mexico. We combine machine learning (ML) to characterise non-linear individual-level patterns, Bayesian spatial models (BSM) to quantify geographic clustering and contextual heterogeneity, and dynamic structural equation modelling (DSEM) to examine candidate pathways and feedbacks linking income with early motherhood. Together, these analyses inform equity-oriented strategies that integrate education, sexual and reproductive health, and social protection.

## Methods

### Design and data sources

A multi-approach, dynamic study was conducted using harmonised microdata from the Mexican Population and Housing Censuses (2000, 2010, and 2020) [48]. The following approaches were selected to capture complementary dimensions: (i) an individual-level ML approach [49] to identify the strongest predictors and nonlinear relationships associated with early motherhood; (ii) BSM [50] at the municipal level to quantify geographic patterns and determinants at the territorial level; and (iii) DSEM [51] to examine potential pathways and lagged relationships through which income is linked to early motherhood. For the individual-level analyses, we used data from the extended (long-form) census questionnaire, which is administered to a representative subsample of the population from each municipality (86% of all municipalities in Mexico, *n* = 2,100). We used an age range of 12–24 years to operationalise early motherhood in a way that could be measured consistently in harmonised long-form census microdata across the 2000, 2010, and 2020 census rounds [42]. This choice was informed by contemporary developmental and social-role evidence indicating that biological maturation, neurocognitive development, and key transitions—including schooling, labour market entry, union formation, and parenthood—often extend into the early twenties [42]. The main analyses were conducted for women aged 12–24 years as a single analytical group, with age included directly in the individual-level models. Accordingly, age-related heterogeneity within this range was addressed through direct adjustment for age rather than by redefining the study outcome as a broader transition to adulthood. The lower bound (12 years) reflects the minimum age for which key individual-level measures were consistently available and harmonisable across census rounds. Accordingly, the unit of observation was women aged 12–24 years at the time of the survey who were enumerated in each census round, yielding 3.6 million records representing 33.4 million women nationwide. After removing observations with missing data on key covariates, the final analytical sample included 3.1 million records, representing 30.7 million women, with an overall missingness rate of 8%. No meaningful differences were observed in study variables between individuals included in the analyses and those excluded because of missing, incorrect, or incomplete data.

For the municipality-level analyses, the sampling frame covered 2,100 officially recognised municipalities, accounting for 86% of all municipalities in Mexico in 2000. To ensure comparability of territorial units over time, we constructed a balanced panel restricted to municipalities with a consistently observed official municipal identifier across the three census rounds; municipalities affected by administrative reconfigurations (e.g., splits/merges that interrupt longitudinal continuity) were excluded at the panel-building stage. After creating a balanced municipal panel, 1,966 municipalities were retained for the spatial and DSEM analyses (missingness = 6%). All microdata were harmonised to ensure consistent variable definitions across census rounds; further details on harmonisation procedures and variable coding are available in Sect.  1 of the Supplementary Material.

### Outcome

Our primary outcome was early motherhood within the 12–24 analytical age band. At the individual level, we generated a binary variable coded as one if a woman aged 12–24 years reported at least one live birth at the time of the census, and zero otherwise. At the municipal level, we calculated the early motherhood prevalence (proportion) for each census year as the number of women aged 12–24 years with one or more live births divided by the total number of women in the same age group within the municipality. These proportions were used both as direct outcome measures (when modelling counts or proportions) and, where appropriate, transformed (e.g., using the complementary log transformation) to meet model assumptions [50].

### Exposure: household income

Although the censuses include a labour-income module (via the extended (long-form) questionnaire), this measure captures only one component of household resources and has been shown to underreport labour earnings relative to specialised income-measurement sources such as ENIGH [52]. Moreover, non-labour income—including public transfers, private transfers and remittances—can represent a substantial share of total resources, particularly among economically vulnerable households [53–55]. To obtain a welfare-relevant measure that is comparable across households with different sizes and compositions, we imputed total quarterly household income (labour + non-labour) per adult equivalent for all census households [56]. We equivalised income to account for household economies of scale and differential needs (rather than using per-capita income, which implicitly assumes equal needs and no scale economies), applying the rescaled modified-OECD equivalence scale—a widely used standard in official income-distribution work and originally proposed for international comparability [56–59]. Specifically, we computed each household’s equivalence factor using the rescaled weights—first adult = 0.67; each additional adult aged ≥ 18 = 0.33 regardless of headship; child aged 14–17 = 0.33; child aged 0–13 = 0.20—and divided total household income by this factor to obtain equivalised income [56]. We expressed income in constant 2018 international dollars (Int-US$ PPP), and imputed it for all census households using a Quantile Random Forest (QRF) regression model [49] trained on recent rounds of Mexico’s National Household Income and Expenditure Survey (ENIGH, in Spanish) for 2000, 2010, and 2020 [60]. ENIGH is a biennial survey that gathers detailed data on quarterly household income and expenditure, as well as the economic activities of household members. It employs a probabilistic, stratified, two-stage cluster sampling design, allowing for the production of representative estimates at both national and state levels, with urban–rural disaggregation [60]. Reference periods were aligned by using ENIGH quarterly income definitions and applying the same price deflation and PPP conversion procedures to all years.

Predictors of imputed income included household composition and dependency ratios; the educational attainment of the household head; and a set of assets (e.g. radio, television, refrigerator, washing machine, car, computer) and housing-quality indicators (e.g. floor and dwelling type, number of rooms, access to basic services—including water, sanitation, electricity—overcrowding, and waste disposal method). Additional predictors included contextual municipal indicators—such as gross domestic product per capita, formal employment rate, and social security coverage—sourced from official statistics.

Each ENIGH round was randomly split into training (70%) and testing (30%) subsamples, with hyperparameters optimised through stratified 10-fold cross-validation to minimise mean absolute error (MAE) [61]. Survey design and expansion factors were incorporated during both training and evaluation (via the Ranger and DALEX interfaces) [49, 61]. Model performance was assessed on the hold-out sample [49, 61], and all estimates account for survey expansion factors.

To address the known tendency of QRF models to bias predictions at the distributional extremes, we examined MAE across income deciles. We adjusted the quantile parameter (tau) in the lowest (deciles 1–3) and highest deciles (decile 10) to reduce systematic over- or under-estimation. Final imputed income values for census households corresponded to the conditional median (tau = 0.5), incorporating these decile-specific adjustments. Municipal-level income estimates were derived as the median imputed per-adult-equivalent household income quarterly within each municipality and census year. Detailed diagnostics, predictor importance, and additional methodological details on the imputation procedure are described in Sect. 2 of the Supplementary Material and in the companion working paper [62]. The QRF was implemented in R 4.4.0 using the ranger package [49].

### Covariates

Covariates were selected based on prior evidence linking socioeconomic and demographic factors to adolescent fertility and its underlying social determinants [9, 18, 23, 33, 63]. We assembled an evidence-informed set of individual, household, and municipal covariates to reduce confounding and to capture key mediating pathways.

At the individual level, covariates included age (years); main activity (student, unpaid domestic work, employed, incapatitated to work, pensioner o retiree, unemployed, and looking for job); marital status (single; married/common-law; separated/divorced/widowed); schooling lag (expected years of schooling by age minus completed years [9]); health insurance status (none, any, multiple); self-reported indigenous language; and illiteracy.

At the household level, we included socioeconomic status (SES) as a complementary measure of longer-run material conditions. We conceptualised income and SES as related but non-equivalent dimensions of household material conditions: income reflects more immediate command over goods and services, whereas SES captures more durable asset- and housing-based conditions [64, 65]. The SES was constructed from a standardised set of asset indicators (radio, television, refrigerator, washing machine, car, computer) and housing-quality indicators (floor and dwelling type, number of rooms, access to basic services—including water, sanitation, and electricity—overcrowding, and waste disposal method). We derived the SES using Multiple Correspondence Analysis (MCA) [66, 67] applied to the 2000 census and then applied the first-dimension loadings to the 2010 and 2020 censuses to preserve temporal comparability. Anchoring the loadings to the 2000 baseline allowed secular improvements in services and housing conditions to shift SES values while preserving a constant interpretation of the SES construct across census rounds. Higher values indicate greater asset ownership and better housing conditions. Further details are provided in Sect. 2 of the Supplementary Material. Additional household-level covariates included characteristics of the household head (age, sex, education, and main activity), household type (nuclear, extended, composite, single-person), demographic dependency ratio, housing type/tenure (owned, rented, other), and an indigenous household indicator as defined by official criteria [68].

At the municipal level, we recorded the official municipal identifier and degree of urbanicity (rural, urban, or metropolitan) as a time-varying municipal characteristic. This measure was intended to capture observable changes in settlement structure across census rounds, but not the full range of peri-urban or metropolitan transformation that may occur over time. The census year was incorporated as a fixed effect to control for secular trends across rounds in both the ML and spatial models. For municipal-level analyses, we computed rates and means for continuous covariates, as well as the proportions of categorical variables most strongly associated with early motherhood, according to the ML model. Data were aggregated at the municipal level using official municipal identifiers provided in the census microdata. Operational definitions and coding schemes are detailed in Sect.  1 of the Supplementary Material.

### Analysis

#### Individual-level machine learning

We first described the characteristics of the study population (women aged 12–24 years) for the pooled sample, based on whether they had experienced early motherhood. Summary statistics, including medians and percentages, were used for this purpose. To identify the most critical risk factors associated with early motherhood, we trained a Random Forest (RF), a widely used and high-performing baseline model in applied health research, as a supervised classification model using 10-fold cross-validation. This method allows the detection of complex, non-linear relationships among a large number of variables. To ensure robustness, the dataset was randomly divided into a training set (70%) and a validation set (30%). Model performance was evaluated using the area under the receiver operating characteristic curve (AUC), accuracy, F1 score (a metric that balances precision and recall, mainly when class imbalance occurs), and precision.

To identify the most influential predictors, we applied an agnostic interpretability approach [69, 70], which estimated variable importance from 50 random subsamples (*n* = 10,000 each) and visualised the marginal effects of key predictors on the probability of early motherhood using partial dependence plots (*n* = 100,000, 95% confidence intervals, CI). The ranger package [49] was used to train the model, and the DALEX package [61] was used to derive performance metrics, variable importance rankings, and partial dependence plots. The final Random Forest model demonstrated strong predictive performance (accuracy = 0.94; AUC = 0.98; F1 = 0.88; precision = 0.88). All estimates account for survey expansion factors. Additionally, we trained RF models to predict motherhood, assess the importance of variables, and examine the predictive relationship of income per adult equivalent in three age groups (under 15 years, 15–19 years, and over 20 years). The results are shown in Figure A15 of Sect. 3.

#### Municipal-level Bayesian spatial modelling

To assess the geographic and temporal variation in early motherhood and identify potential high-risk areas, we implemented a series of spatial and spatiotemporal models. First, we calculated the Local Moran’s I statistic and both direct and corrected Local Indicators of Spatial Association (LISA), adjusted for the top 10 predictors identified by the machine learning model, to detect significant spatial clustering and dependence [71–73]. We used a K-nearest-neighbours spatial weights matrix (K = 3) as a pragmatic baseline neighbourhood definition, and assessed robustness to alternative neighbourhood sizes (K = 2–5) in sensitivity analyses.

Next, we fitted a spatiotemporal Bayesian model using Integrated Nested Laplace Approximation (INLA) [50]. The model assumed a beta-binomial distribution for the dependent variable to account for the overdispersion typically observed in proportion data. The spatial structure was specified through a binary adjacency matrix based on municipal contiguity, constructed on the harmonised set of municipalities included in the balanced panel, ensuring consistency of geographic boundaries across census years. The Besag–York–Mollié 2 (BYM2) component captured spatial autocorrelation, while a first-order random walk (RW1) term modelled smooth temporal evolution.

The ten most influential predictors identified by the RF model were included as fixed effects, but those that worsened the model fit were excluded from the final model. Model performance and fit were compared using the deviance information criterion (DIC), Watanabe–Akaike information criterion (WAIC), and marginal log-likelihood. The beta–binomial specification demonstrated good model fit (DIC = − 29,686.86; WAIC = − 28,323.88; marginal log-likelihood = 14,279.42), with an effective complexity of 78.36 parameters. Spatially structured and unstructured random effects, precision estimates, and posterior summaries of fixed effects (means and 95% credible intervals) are reported in the Results section. High-risk areas were identified by mapping the posterior deciles of the spatial effects.

#### Municipality-level dynamic structural equation modelling

To examine the dynamic relationships between socioeconomic context and early motherhood at the municipal level, we implemented DSEM [51]. This approach allows the assessment of how variables co-vary and are temporally related across measurement occasions, capturing both immediate and lagged effects (short-, medium-, and long-term). The final model demonstrated a substantial improvement in fit compared to the initial specification, both in terms of likelihood and parametric efficiency. While the first model (with the ten most important predictors for the RF model) estimated more than 120 parameters and had a log-likelihood of -99,262 and an AIC of 198,812, the refined model reduced the number of parameters to 49 without sacrificing explanatory power, achieving a log-likelihood of -51,658.8 and an AIC of 103,415.7. This reduction of more than 60% in the number of parameters was accompanied by a marked decrease in the AIC (ΔAIC ≈ 95,396), consistent with improved fit relative to model complexity and supporting the selection of a more parsimonious specification.

Furthermore, the log-posterior was closely aligned with the log-likelihood, suggesting limited prior influence under the chosen specifications and that model fit is largely driven by the empirical likelihood. Taken together, these metrics confirm that the final model offers a better balance between fit and complexity for the set of candidate specifications considered. The drift parameters identified two types of temporal dependence: autoregressive effects, which represent how each variable develops based on its own past values, and cross-lagged associations, describing how one variable is related to another in subsequent periods. Spatial autocorrelation was accounted for through the BYM2 component, while smooth temporal trends were modelled via a first-order RW1 term. With only three measurement occasions, prior methodological work suggests that parsimonious DSEM specifications can still estimate first-order autoregressive and cross-lagged parameters with T = 3; we therefore treat these parameters as descriptive summaries of temporal co-movement, not as definitive causal effects [74].

Model estimation relied on Bayesian inference using Markov Chain Monte Carlo (MCMC) methods [51], a robust computational framework for estimating complex dynamic relationships. Several strategies were implemented to ensure reliability and convergence: (i) multiple chains were used to verify the stability and consistency of posterior estimates; (ii) convergence diagnostics confirmed that parameter estimates achieved stationary distributions; and (iii) standardised statistical criteria were applied to assess model accuracy and adequacy.

The final model was constructed incrementally, adding variables based on their significance. This approach helped identify different patterns of temporal relationships, including positive reinforcement (where effects grow over time), negative reinforcement (where effects diminish), and compensatory relationships (where interactions between variables moderate individual effects).

Model evaluation using standardised fit criteria [51] confirmed its capacity to adequately represent the underlying temporal and spatial dependencies in the data. This methodological strategy enabled not only the identification of contemporaneous associations but also the disentangling of the complex network of temporal feedbacks linking socioeconomic conditions and early motherhood at the municipal level.

#### Sensitivity analyses

Alternative numbers of nearest neighbours (K = 2, 3, 4, and 5) were considered to assess the robustness of the LISA and Moran’s Index results to alternative definitions of local neighbourhoods and potential changes in spatial connectivity over time. As part of the sensitivity analysis, we also evaluated different initialisations of parameters for the DSEM model. Using the final model as the baseline, we tested variations in key parameter initialisations by adjusting their starting values in steps of five-tenths (e.g., the effect of income on marital status initialised at − 0.1, − 0.5, − 1.0, − 1.2, and 2.0), while preserving the theoretically expected direction of the impact (positive or negative). The full results are shown in Sect.  6 of the Supplementary Material.

## Results

### Descriptive results

Over the two decades analysed, early motherhood remained predominantly concentrated in socioeconomically marginalised households among women aged 12 to 24 (Table 1). The unadjusted prevalence of early motherhood decreased only slightly, from 21.46% in 2000 to 20.98% in 2010 and to 19.31% in 2020, with an overall average of 20.70% across the period. Although some indicators improved modestly over time, gaps between mothers (women aged 12–24 with ≥ 1 live birth) and non-mothers persisted across census rounds. Unadjusted educational attainment among mothers rose significantly, from 39.82% who completed high school in 2000 to 43.89% in 2010 and 45.06% in 2020. The schooling lag decreased among mothers from 4.28 years (2000) to 2.12 years (2020), and among non-mothers from 1.24 to 0.52 years. The schooling attainment of household heads increased among mothers (from 30.55% who completed secondary schooling level in 2000 to 38.25% in 2020), while among non-mothers it rose from 19.09% in 2000 to 32.74% in 2020. Similarly, the average age of household heads among mothers increased from 34.47 to 40.08 years, yet remained consistently younger than that of non-mothers (from 44.79 to 46.8 years).

**Table 1:** Main characteristics of women aged 12–24 years in assessment of early motherhood in Mexico 2000–2020

	Estimated percentage or mean among Mexican adolescents (95% CI)							
	2000		2010		2020		Overall	
	With early motherhood	Without early motherhood	With early motherhood	Without early motherhood	With early motherhood	Without early motherhood	With early motherhood	Without early motherhood
N (%)	1,796,919 (21.46)	6,574,677 (78.54)	2,374,659 (20.98)	8,533,446 (79.02)	2,185,491 (19.31)	9,130,102 (80.69)	6,357,069 (20.70)	24,238,225 (79.30)
**Individuals**								
Age, avrg	21.45 [21.44—21.47]	16.93 [16.92—16.94]	21.27 [21.25—21.29]	16.98 [16.96—17.00]	21.46 [21.44—21.47]	17.12 [17.11—17.14]	21.39 [21.38, 21.39]	17.02 [17.01, 17.02]
Schooling level (%)								
Nothing	<1%	<1%	1.62 [1.6—1.63]	<1%	<1%	<1%	<1%	<1%
Primary	35.94 [35.87—36.01]	26.61 [26.58—26.65]	20.27 [20.21—20.32]	21.76 [21.73—21.79]	11.45 [11.41—11.5]	15.12 [15.09—15.14]	21.67 [21.63—21.7]	20.57 [20.56—20.59]
Secondary	39.82 [39.75—39.89]	39.5 [39.47—39.54]	43.89 [43.83—43.96]	37.79 [37.75—37.82]	45.06 [44.99—45.13]	35.69 [35.66—35.72]	43.14 [43.1—43.18]	37.46 [37.44—37.48]
High school or over	24.03 [23.96—24.09]	33.71 [33.67—33.74]	34.23 [34.16—34.29]	39.61 [39.58—39.65]	43.42 [43.35—43.48]	49.05 [49.02—49.08]	34.5 [34.47—34.54]	41.57 [41.55—41.59]
Schooling lag, avrg	4.28 [4.26—4.30]	1.24 [1.23—1.25]	3.2 [3.17—3.22]	<1yr	2.12 [2.1—2.14]	<1yr	3.13 [3.12, 3.14]	<1yr
Parity (%)								
Primiparous	61.24 [61.17—61.31]	NA	64.27 [64.21—64.33]	NA	67.62 [67.55—67.68]	NA	64.56 [64.52—64.6]	NA
Two	10.27 [10.22—10.31]	NA	8.84 [8.81—8.88]	NA	6.39 [6.36—6.42]	NA	27.04 [27—27.07]	NA
Three or over	28.5 [28.43—28.56]	NA	26.89 [26.83—26.95]	NA	26 [25.94—26.05]	NA	8.4 [8.38—8.42]	NA
Health insurance (%)	41.4 [41.33—41.47]	43.72 [43.68—43.75]	64.87 [64.81—64.93]	62.54 [62.51—62.58]	74.59 [74.53—74.64]	73.87 [73.85—73.9]	60.28 [60.25—60.3]	60.04 [60—60.6]
Mixed health insurance (%)	<1%	<1%	<1%	<1%	<1%	1.53 [1.53—1.54]	<1%	<1%
Marital status (%)								
Married/free civil union	85.45 [85.4—85.5]	6.94 [6.92—6.96]	78.84 [78.79—78.9]	7.34 [7.32—7.36]	75.23 [75.17—75.29]	6.3 [6.28—6.31]	79.47 [79.44—79.5]	6.84 [6.83—6.85]
Separated/Divorcee/Widower	5.86 [5.83—5.90]	<1%	8.17 [8.14—8.21]	<1%	11.76 [11.72—11.81]	<1%	8.75 [8.73—8.78]	<1%
Single	8.69 [8.65—8.73]	92.89 [92.87—92.91]	12.98 [12.94—13.03]	92.39 [92.38—92.41]	13 [12.96—13.05]	93.47 [93.46—93.49]	11.78 [11.75—11.8]	92.93 [92.92—92.94]
Activity condition (%)								
Student	1.59 [1.57—1.61]	46.85 [46.81—46.89]	3.57 [3.55—3.6]	61.56 [61.52—61.59]	3.81 [3.79—3.84]	64.9 [64.87—64.94]	3.1 [3.08—3.11]	58.83 [58.81—58.85]
Employed	25.71 [25.64—25.77]	26.81 [26.78—26.85]	28.56 [28.5—28.61]	19.84 [19.82—19.87]	33.45 [33.38—33.51]	19.96 [19.93—19.98]	29.43 [29.4—29.47]	21.78 [21.76—21.79]
Domestic work	62.26 [62.19—62.33]	11.72 [11.7—11.75]	65.44 [65.38—65.5]	14.12 [14.1—14.14]	59.14 [59.07—59.2]	9.27 [9.25—9.28]	62.37 [62.34—62.41]	11.64 [11.63—11.65]
Other	10.44 [10.39–10.48]	14.61 [14.58–14.64]	2.43 [2.41–2.45]	4.48 [4.47–4.5]	3.6 [3.58–3.63]	5.87 [5.86–5.89]	5.1 [5.08–5.11]	7.75 [7.74–7.76]
Speak an indigenous language	4.98 [4.95—5.01]	3.61 [3.59—3.62]	6.23 [6.2—6.26]	4.85 [4.83—4.86]	5.58 [5.55—5.61]	3.51 [3.5—3.52]	5.65 [5.64—5.67]	4.01 [4—4.01]
**Household head**								
Women (%)	13.29 [13.24—13.34]	17.94 [17.91—17.97]	19.77 [19.72—19.82]	21.84 [21.82—21.87]	28.48 [28.42—28.54]	29.85 [29.82—29.88]	20.93 [20.9—20.96]	23.8 [23.79—23.82]
Age, avrg	34.47 [34.39—34.55]	44.79 [44.76—44.82]	39.42 [39.3—39.55]	46.54 [46.48—46.6]	40.08 [39.97—40.19]	46.8 [46.76—46.84]	38.25 [38.21, 38.28]	46.16 [46.15, 46.18]
Schooling level (%)								
Nothing	0.43 [0.42—0.44]	0.52 [0.51—0.52]	7.96 [7.93—8]	7.76 [7.74—7.78]	0.30 [0.29—0.31]	0.24 [0.23—0.24]	3.20 [3.19—3.21]	2.96 [2.95—2.97]
Primary	47.67 [47.6—47.74]	52.93 [52.89—52.97]	38.26 [38.2—38.32]	36.84 [36.8—36.87]	33.38 [33.32—33.44]	28.48 [28.46—28.51]	39.24 [39.2—39.28]	38.06 [38.04—38.08]
Secondary	30.55 [30.48—30.62]	19.09 [19.06—19.12]	30.35 [30.3—30.41]	24.23 [24.2—24.25]	38.25 [38.19—38.32]	32.74 [32.71—32.77]	33.12 [33.09—33.16]	26.04 [26.02—26.06]
High school or over	21.35 [21.29—21.41]	27.46 [27.43—27.50]	23.42 [23.37—23.48]	31.18 [31.15—31.21]	28.07 [28.01—28.13]	38.54 [38.51—38.57]	24.44 [24.4—24.47]	32.94 [32.92—32.96]
Activity condition (%)								
Unemployed	3.6 [3.58—3.63]	4.56 [4.54—4.57]	2.38 [2.36—2.4]	2.46 [2.45—2.47]	2.25 [2.24—2.27]	2.15 [2.14—2.16]	2.68 [2.67—2.7]	2.91 [2.91—2.92]
Employed	90.05 [90.01—90.1]	87.93 [87.91—87.96]	85.68 [85.64—85.73]	86.23 [86.21—86.25]	82.54 [82.49—82.6]	84.67 [84.65—84.69]	85.84 [85.81—85.87]	86.1 [86.09—86.12]
Domestic work	4.62 [4.59—4.65]	4.8 [4.78—4.81]	7.26 [7.23—7.29]	6.05 [6.04—6.07]	10.77 [10.73—10.81]	7.76 [7.74—7.77]	7.72 [7.7—7.74]	6.35 [6.34—6.36]
Other	1.72 [1.7–1.74]	2.72 [2.7–2.73]	4.68 [4.65–4.71]	5.25 [5.24–5.27]	4.43 [4.4–4.46]	5.42 [5.41–5.43]	3.76 [3.74–3.77]	4.63 [4.62–4.64]
**Household**								3.99 [3.97-4]
Type (%)								
Unipersonal	<1%	<1%	<1%	<1%	<1%	<1%	<1%	<1%
Nuclear	57.34 [57.27—57.42]	69.64 [69.6—69.67]	41.38 [41.32—41.44]	66.23 [66.2—66.26]	37.81 [37.74—37.87]	67.29 [67.26—67.33]	44.66 [44.62—44.7]	67.56 [67.54—67.57]
Extended	41.06 [40.99—41.13]	28.07 [28.03—28.10]	56.94 [56.87—57]	31.42 [31.39—31.45]	61.08 [61.02—61.15]	30.68 [30.65—30.71]	53.87 [53.84—53.91]	30.23 [30.21—30.25]
Composite	1.39 [1.38—1.41]	1.73 [1.72—1.74]	1.49 [1.47—1.51]	1.82 [1.81—1.83]	<1%	1.24 [1.23—1.25]	1.28 [1.27—1.29]	1.58 [1.57—1.58]
Indigenous (%)	7.69 [7.65—7.73]	7.40 [7.38—7.42]	10.08 [10.04—10.12]	9.81 [9.79—9.83]	9.46 [9.43—9.5]	7.85 [7.83—7.87]	9.19 [9.17—9.21]	8.42 [8.41—8.43]
Number Bedrooms (avrg)	1.94 [1.94—1.95]	2.46 [2.46—2.47]	2.05 [2.04—2.06]	2.43 [2.42—2.44]	2.14 [2.13—2.15]	2.47 [2.46—2.47]	2.05 [2.05, 2.05]	2.45 [2.45, 2.45]
Overcrowding (%)	59.75 [59.67—59.82]	35.32 [35.29—35.36]	58.00 [57.94—58.06]	30.77 [30.73—30.8]	51.93 [51.87—52]	22.99 [22.96—23.01]	56.41 [56.37—56.45]	29.07 [29.05—29.09]
Number of adult equivalents (AE) (avrg)	1.76 [1.76—1.77]	2.01 [2.01—2.01]	1.92 [1.91—1.92]	1.94 [1.94—1.94]	1.85 [1.84—1.85]	1.83 [1.83—1.83]	1.85 [1.85—1.85]	1.92 [1.92—1.92]
Demographic dependence rate (%)	3.17 [3.16—3.18]	3.14 [3.13—3.15]	3.15 [3.14—3.16]	3.14 [3.13—3.15]	3.00 [2.99—3.01]	2.93 [2.92—2.93]	3.10 [3.10—3.11]	3.06 [3.06—3.06]
Socioeconomic status (%)								
Lowest	16.01 [15.95—16.06]	11.14 [11.12—11.17]	8.98 [8.94—9.02]	6.04 [6.03—6.06]	11.70 [11.66—11.74]	6.59 [6.57—6.61]	11.9 [11.88—11.93]	7.63 [7.62—7.64]
Low	26.44 [26.37—26.50]	16.87 [16.84—16.90]	20.89 [20.84—20.95]	12.48 [12.46—12.51]	21.66 [21.61—21.72]	12.33 [12.31—12.35]	22.73 [22.69—22.76]	13.62 [13.6—13.63]
Middle	26.49 [26.43—26.56]	21.17 [21.14—21.20]	27.48 [27.42—27.53]	19.03 [19—19.06]	29.75 [29.69—29.81]	19.59 [19.57—19.62]	27.98 [27.95—28.02]	19.82 [19.81—19.84]
High	20.74 [20.68—20.8]	23.45 [23.42—23.48]	24.58 [24.53—24.64]	23.85 [23.82—23.88]	20.34 [20.28—20.39]	22.72 [22.69—22.74]	22.04 [22—22.07]	23.31 [23.3—23.33]
Highest	10.32 [10.28—10.37]	27.37 [27.34—27.40]	18.07 [18.02—18.12]	38.59 [38.56—38.63]	16.55 [16.5—16.6]	38.77 [38.74—38.8]	15.36 [15.33—15.39]	35.61 [35.6—35.63]
Income per AE (avrg)^a^	1.66 [1.65—1.67]	2.79 [2.78—2.80]	1.36 [1.35—1.37]	2.22 [2.22—2.23]	1.73 [1.69.1.77]	1.86 [1.86—1.87]	1.38 [0.05 — 38.49]	2.24 [0.03 — 82.55]
Income per capita (avrg)^a^	0.62 [0.67—0.62]	1.08 [1.08—1.09]	0.25 [0.49—0.50]	0.87 [0.87—0.88]	0.41 [0.41— 0.41]	0.74 [0.74—0.75]	0.51 [0.02 — 14.72]	0.89 [0.01 — 49.78]
Total household income^a^	2.82 [2.8—2.84]	5.12 [5.1—5.14]	2.49 [2.48—2.15]	3.92 [3.91—3.93]	2.04 [2.03—2.04]	3.14 [3.14 —3.15]	2.43 [0.08 — 98.58]	3.96 [0.06 — 111.61]
Highest school level (%)								
Nothing	24.25 [24.19—24.32]	42.22 [42.18—42.26]	15.54 [15.5—5.59]	21.37 [21.34—21.4]	22.24 [22.19—22.3]	44.58 [44.55—44.62]	20.31 [20.28—20.34]	35.77 [35.75—35.79]
Primary	<1%	NCR	<1%	<1%	NCR	<1%	<1%	<1%
Secondary	16.95 [16.68—17.00]	8.04 [8.02—8.06]	9.55 [9.51—9.59]	6.21 [6.19—6.22]	3.7 [3.68—3.73]	1.68 [1.67—1.69]	9.63 [9.61—9.65]	5 [4.99—5.01]
High school or over	58.77 [58.7—58.85]	49.74 [49.7—49.78]	74.85 [74.79—74.9]	72.41 [72.38—72.44]	74.05 [73.99—74.11]	53.73 [53.7—53.77]	70.03 [69.99—70.07]	59.23 [59.21—59.25]
**Place of residence/Localy population size**								
Rural (%)	18.34 [18.28—18.39]	16 [15.97—16.03]	22.53 [22.48—22.58]	21.25 [21.22—21.27]	22.97 [22.91—23.03]	17.21 [17.19—17.23]	21.5 [21.46—21.53]	18.3 [18.29—18.32]
Urban (%)	29.16 [29.09—29.22]	27.78 [27.75—27.81]	32.23 [32.17—32.29]	29.69 [29.66—29.72]	34.16 [34.1—34.22]	31.02 [30.99—31.05]	32.02 [31.99—32.06]	29.67 [29.65—29.69]
Metropilitan (%)	52.51 [52.44—52.58]	56.22 [56.18—56.26]	45.24 [45.18—45.31]	49.07 [49.03—49.10]	42.87 [42.8—42.94]	51.77 [51.73—51.8]	46.48 [46.44—46.52]	52.03 [52.01—52.05]
Socioeconomic status Region (%)								
Lowest	8.03 [7.99—8.07]	7.47 [7.45—7.49]	10.6 [10.56—10.64]	9.94 [9.92—9.96]	10.19 [10.15—10.23]	8.49 [8.47—8.5]	9.73 [9.71—9.75]	8.72 [8.71—8.73]
2	19.44 [19.38—19.50]	19.14 [19.11—19.17]	20.15 [20.09—20.2]	20.06 [20.04—20.09]	19.76 [19.71—19.82]	18.54 [18.52—18.57]	19.81 [19.78—19.85]	19.24 [19.22—19.26]
3	11.2 [11.15—11.25]	11.86 [11.83—11.88]	12.86 [12.82—12.91]	13.07 [13.05—13.09]	14.38 [14.34—14.43]	12.68 [12.65—12.70]	12.92 [12.89—12.94]	12.59 [12.58—12.61]
4	24.96 24.9—25.03]	24.6 [24.56—24.63]	24.25 [24.2—24.3]	24.01 [23.98—24.04]	23.65 [23.59—23.7]	25.32 [25.3—25.35]	24.24 [24.21—24.28]	24.66 [24.65—24.68]
5	13.68 [13.63—13.73]	11.03 [11—11.05]	13.08 [13.04—13.13]	11.08 [11.06—11.10]	12.47 [12.42—12.51]	12.4 [12.38—12.42]	13.04 [13.01—13.07]	11.56 [11.55—11.58]
6	13.85 [13.8—13.90]	15.09 [15.06—15.11]	13.21 [13.16—13.25]	14.12 [14.1—14.14]	14.96 [14.92—15.01]	15.27 [15.25—15.29]	13.99 [13.97—14.02]	14.82 [14.8—14.83]
Highest	8.84 [8.8—8.88]	10.82 [10.8—10.84]	5.85 [5.82—5.88]	7.71 [7.69—7.73]	4.59 [4.56—4.62]	7.30 [7.28—7.32]	6.26 [6.24—6.28]	8.41 [8.39—8.41]

Note: ^a^Expressed in thousands of constant 2018 international dollars, adjusted for purchasing power parity (PPP) for household final consumption expenditure (PPP-IntUS$). NA: Not available. NCR: No cases reported. AE: Number of adult equivalents. 95% CI: 95% Confidence Interval

Family and living conditions also differed across groups and over time. In all census rounds, mothers were considerably less likely to live in nuclear households (57.34% in 2000, 41.38% in 2010, and 37.81% in 2020), compared to 69.64%, 66.23%, and 67.29% among non-mothers. Overcrowding, although generally decreasing, remains highly prevalent among mothers, falling from 59.75% in 2000 to 51.93% in 2020, although it is still much higher than among non-mothers (from 35.32% to 22.99%). Household size, measured in adult equivalents, remained almost stable (1.76 → 1.92 → 1.85), but smaller than among non-mothers (2.01 → 1.94 → 1.83). Mothers were more likely to live in extended or composite households than non-mothers across census rounds (Table 1).

Socioeconomic stratification also persisted: the proportion of mothers in the lowest socioeconomic quintile decreased from 16.00% in 2000 to 11.7% in 2020, but remained higher than that of non-mothers (from 11.14% to 6.59%). Across years, mothers remained over-represented in the lowest SES quintile relative to non-mothers.

**Fig. 1:**
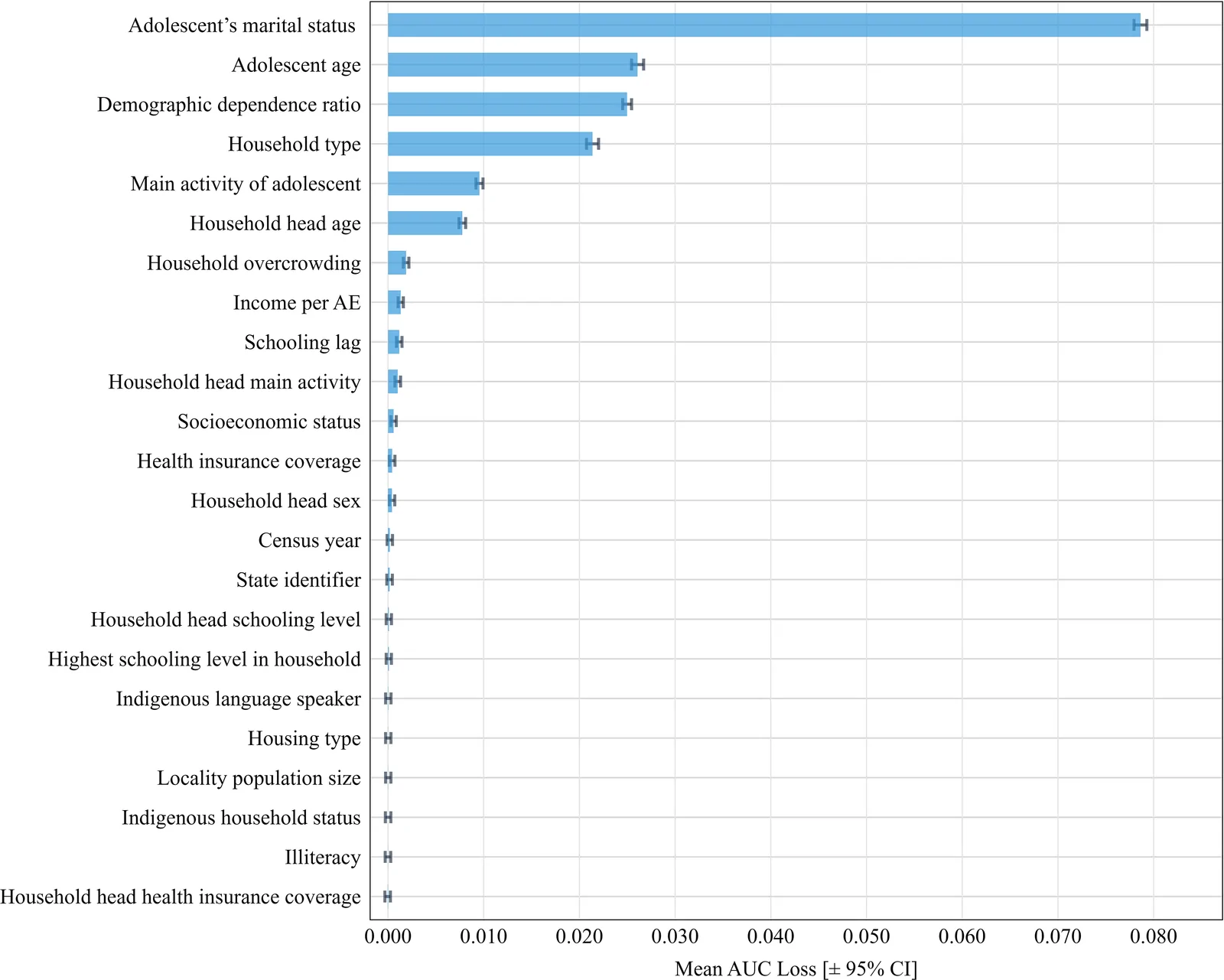
Predictors of early motherhood by importance (random forest model) Note: The bars show the average loss of AUC when permuting each variable (B = 50 permutations) with 95% confidence intervals (CI). AE: Number of adult equivalents, AUC: Area under the curve)

Figure 1 illustrates the relative significance of variables in the RF classification model. The five most influential features for classifying early motherhood status are marital status, the respondent’s age, the demographic dependency ratio, household type, and the respondent’s main activity. Quarterly income per adult equivalent ranks as the eighth most important feature in the classification, and four predictors demonstrated negligible classification importance, with median importance close to zero and 95% CI overlapping zero. Within the 12–24 age range, Figure A13 (Sect.  3 of the Supplementary Material) reports age-stratified RF models (< 15, 15–19, ≥ 20), in which feature importance differs across age groups.

**Fig. 2:**
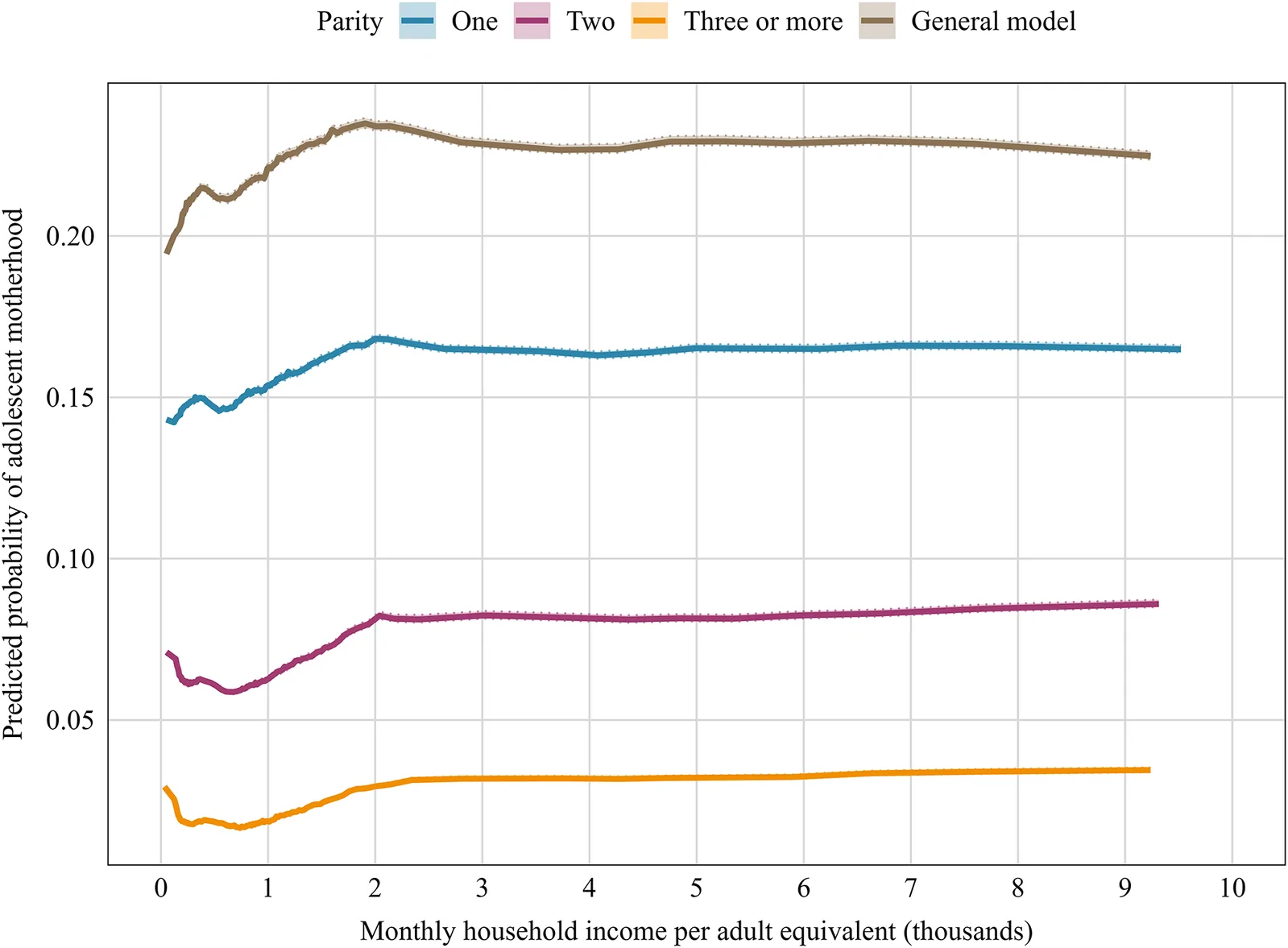
Probability of early motherhood along the adult equivalent income domain. Note: The graph displays how the predicted probability—estimated using the random forest model—of classifying an adolescent as a teen mother changes with variations in household income measured in adult equivalents. The brown line represents the base model. The blue, red, and orange lines show the corresponding changes in the predicted probability for teen mothers with one child, two children, and three or more children, respectively

The partial dependence plots showed a non-linear, inverse L-shaped relationship between quarterly income and the likelihood of early motherhood (Fig. 2). The model-estimated probability rose from 0.19 to 0.23 as income increased up to about Int-US$ PPP 1,760 per equivalent adult, then levelled off and fell. Additional charts (see Sect.  3 of the Supplementary Material) showed higher model-estimated probabilities among those who were married, in a free civil union, or previously separated/divorced, among those living in extended or composite households, and at higher dependency ratios. We also plotted the relationship between the odds of having one, two, or three or more live births (parity) as a function of income using additional models. The parity-1 model showed a pattern similar to the overall model.

In contrast, the models for two children and for three or more children showed a U-shaped association at very low income levels, followed by a gradual flattening as income increases. Across parity-specific models, income’s classification importance declined with increasing parity, becoming negligible for third or higher-order births (Figures A10-12 in Sect.  3 of the Supplementary Material).

### Municipality-level geospatial results

**Fig. 3:**
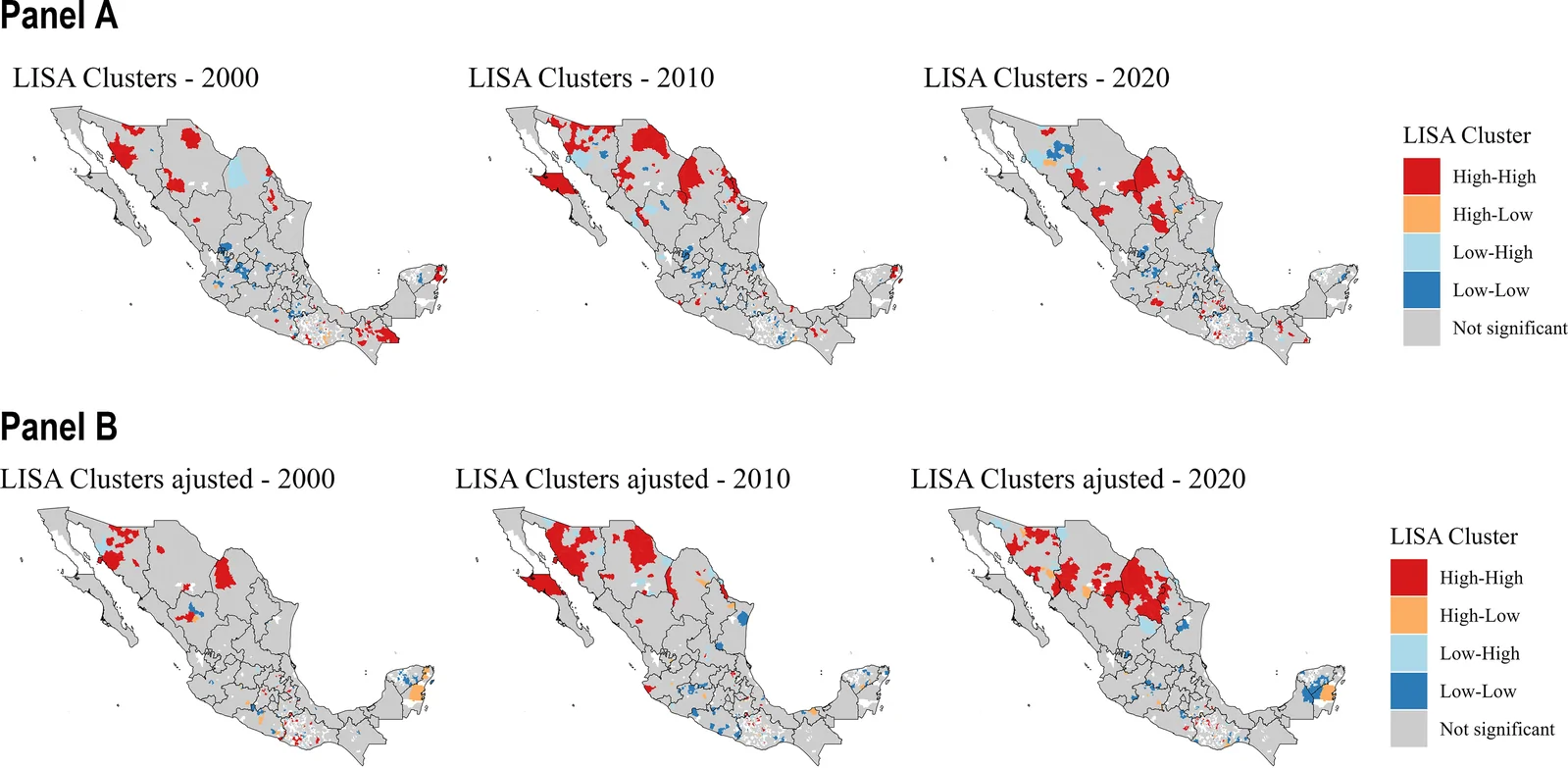
Crude (Panel **A**) and corrected (Panel **B**) LISA clusters for years 2000, 2010 and 2020 in early motherhood rates at the municipal level. Note: The map presents, at the municipal level, the LISA-identified clusters of early motherhood rates for 2000, 2010, and 2020. Panel **A** displays clusters—ranging from high–high to low–low—based on unadjusted rates. Panel **B** presents the corresponding clusters for rates adjusted using the ten most important predictors identified by the random forest model. High–high clusters denote municipalities with high rates surrounded by others with similarly high rates; high–low clusters indicate municipalities with high rates surrounded by municipalities with low rates; low–high clusters reflect municipalities with low rates surrounded by high-rate municipalities; and low–low clusters represent municipalities with low rates surrounded by municipalities with similarly low rates

LISA analyses revealed clear spatial clustering patterns, with significantly high–high clusters concentrated in northern Mexico and low–low clusters in several central and southern regions, consistent across census years. For the initial results, we found moderate Moran indices with statistical significance for each of our three years: for 2000, the index was 0.19 (*p* < 0.05); for 2010, it was 0.29 (*p* < 0.05); and for 2020, it was 0.33 (*p* < 0.05). In Fig. 3 panel A, we display the results of a LISA cluster analysis to identify municipalities with geospatial patterns. After adjusting for the top 10 predictors identified by the machine learning model, Moran’s I coefficients remained significant across all years (2000: I-Moran = 0.12, *p* < 0.05; 2010: I-Moran = 0.28, *p* < 0.05; 2020: I-Moran = 0.24, *p* < 0.05). The clusters identified via the LISA model, adjusted for the top ten predictors, are shown in Fig. 3, panel B.

Spatial components indicated moderate autocorrelation, with structured effects precision of 100.85 (95% CI: 74.84–130.41) and unstructured effects of 819.85 (95% CI: 433.15–1539.28). Temporal RW1 effects were estimated with RW1 precision = 8106.03 (95% CI: 316.21–39759.76).

**Fig. 4:**
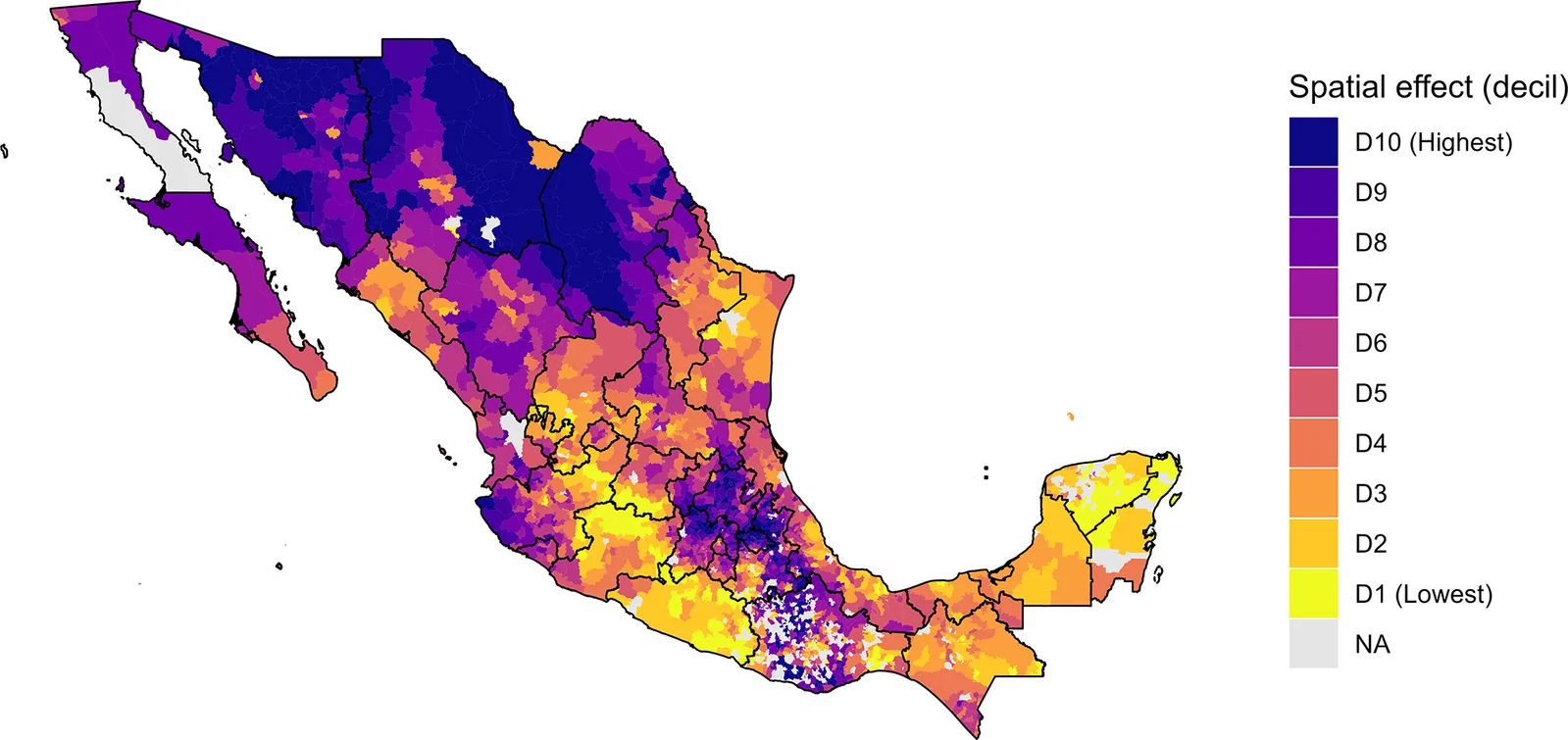
Deciles of average spatial effects found by the INLA model by municipality for 2000–2020. Note: The map shows, at the municipal level, the spatial effect estimated by the INLA model for predicting early motherhood rates. Municipalities in the highest deciles (purple) indicate areas where the spatial component plays a dominant role in the prediction. Conversely, municipalities in the lowest deciles (yellow) represent areas where the socioeconomic variables included in the model were sufficient to achieve accurate predictions with minimal reliance on the spatial structure

Figure 4 illustrates the deciles of the estimated mean spatial effects per municipality over the entire period considered. Municipalities with high residual spatial effects (purple in Fig. 4) were those where area-level (territorial) factors remained strongly associated with early motherhood rates, even after considering socioeconomic covariates. Conversely, municipalities with low deciles of residual spatial effects (yellow in Fig. 4) were those where the socioeconomic covariates considered were sufficient to predict birth rates without territory being a significant factor.

This model revealed that a higher proportion of married women (β = 4.651; 95% CI: 4.566–4.735), extended and composite families (β = 0.125; 95% CI: 0.067–0.183), and a lower level of schooling (β = 0.088; 95% CI: 0.063–0.113) were positively associated with municipal early motherhood rates (Table 2). A later census year (β = 0.133; 95% CI: 0.083–0.190) also showed a positive effect. Negative associations were observed in the proportion of adolescents not attending school (β = -0.177; 95% CI: -0.237 to -0.117) and the mean household head age (β = -0.018; 95% CI: -0.029 to -0.007). Socioeconomic variables, including income from AE (β = 0.004; 95% CI: -0.011–0.018) and mean socioeconomic status (β = 0.022; 95% CI: -0.032–0.075), did not show statistically significant effects at the municipal level, as their credibility intervals included zero. Additionally, in Sect.  4 of the Supplementary Material, we present descriptive statistics by state of the spatial effects identified.

**Table 2:** Mixed space-time model predicting early maternity rates by municipality for the entire period

Parameter	Posterior Mean	Mode
Fixed effects		
Intercept	-2.62 [-2.67— -2.56]	-2.62
Year	0.13 [0.08—0.19]	0.13
Proportion of married adolescents	4.65 [4.56—4.73]	4.65
Income per AE	0.004 [-0.01—0.01]	0.004
Extended or composite families	0.12 [0.06—0.18]	0.12
Schooling lag	0.08 [0.06—0.11]	0.08
Mean socioeconomic status	0.02 [-0.03—0.07]	0.02
Proportion of adolescents not attending school	-0.17 [-0.23— -0.11]	-0.17
Demographic dependency ratio	0.03 [0.02—0.04]	0.03
Mean household head age	-0.01 [-0.02— -0.007]	-0.01
*Random effects (precision)*		
*Spatial effect (IID component)*	819.85 [433.15—1539.28]	656.4
*Spatial effect (structured component)*	100.85 [74.84—130.41]	99.64
*Temporal effect (RW1)*	8106.03 [316.21—39759.76]	766.9
*Model fit criteria*		
*DIC*	-29735.26	
*WAIC*	-26791.24	
*Marginal log-likelihood*	14296.36	

Note: DIC=Deviance Information Criterion; WAIC=Watanabe-Akaike Information Criterion. The model included structured spatial (BYM model) and temporal (RW1 model) random effects. The “Precision for the beta observations” hyperparameter had a mean of 276.92 (SD=5.86). The effective number of parameters was 16.49 for DIC and 1652.36 for WAIC. AE: Number of adult equivalents

### Dynamic structural results

**Fig. 5:**
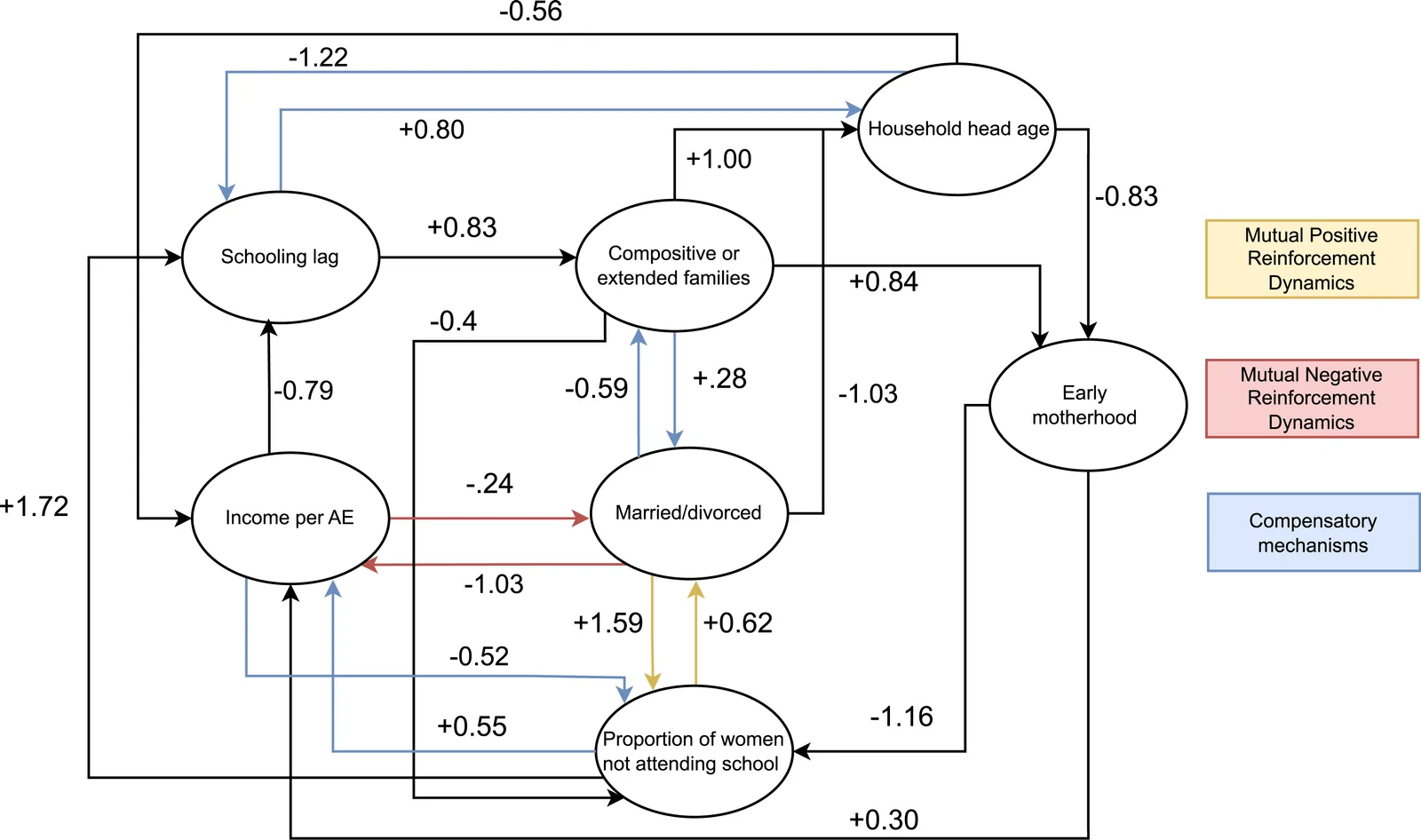
Final dynamic structural model used to predict full-term early motherhood. Note: Unidirectional arrows between variables indicate significant predictions of one variable in state t on another variable in state t + 1. Bidirectional arrows denote one of three possible dynamics: (**a**) positive reinforcement, in which the effect of one variable feeds back and intensifies over time; (**b**) negative reinforcement, where the effect between variables weakens over time; and (**c**) compensatory dynamics, in which variables interact over time in ways that moderate each other’s individual effects. AE: Number of adult equivalents

The DSEM analysis indicated that the direct path between adult-equivalent income and early motherhood was not statistically significant. Figure 5 shows the best-fitting model, where arrows represent one-period lagged relationships (from time t to t + 1). Specifically, (i) a negative reinforcing cycle: income at time t was negatively related to the proportion of women who were married or divorced at time t + 1, which was in turn negatively related to income at time t + 1. (ii) A positive reinforcing cycle: the proportion of married/divorced women at time t was positively related to the proportion of women not attending school at time t + 1, which then was positively related to the proportion of married/divorced women at time t + 1. (iii) Stabilising mechanisms: three pairs of variables showed homeostatic regulation—the proportion of married/divorced women and the proportion of extended or composite families; schooling lag and household head age; and the proportion of women not attending school and average income per adult equivalent. Early motherhood was most strongly related to the average age of the household head and the proportion of extended or composite families. Although the model allowed for a bidirectional relationship between early motherhood and average income per adult equivalent, these paths were not statistically significant. Residual covariance matrices (see Sect.  5 of the Supplementary Material) showed moderate unexplained variance.

## Discussion

This study shows that early motherhood (ages 12–24) in Mexico remained socially patterned across 2000–2020. Despite twenty years of economic and social change, the prevalence of early motherhood fell only slightly—from 21.46% in 2000 to 19.31% in 2020—indicating limited aggregate change in early reproductive transitions among young women aged 12–24 years at each census. Although several socioeconomic indicators improved, gaps between mothers and non-mothers persisted across census rounds. Early motherhood co-occurred with lower schooling attainment, economic insecurity, and higher household dependence, consistent with structural disadvantage shaping life-course opportunities. Income did not emerge as a stand-alone correlate; rather, it clustered with education, union formation, and institutional conditions that organise access to schooling, sexual and reproductive health (SRH) services, and social protection.

Early motherhood remained concentrated among socioeconomically disadvantaged households, where young women typically experience lower schooling attainment, higher dependency ratios, and poorer living conditions [6, 9, 18]. The association between income and early motherhood followed a non-linear inverse L-shaped pattern [5, 9, 17]. The model-estimated probability increased from approximately 0.19 to 0.25 as quarterly household income rose to around Int-US$ PPP 1,760 per adult equivalent, then stabilised and declined slightly at higher income levels. While previous literature has reported non-monotonic associations between income and adolescent childbearing [75–77], the stabilisation rather than a continuous decline at higher income levels warrants attention.

Several factors may help interpret this pattern. First, income was imputed rather than directly observed; prediction-based income measures can be more compressed than observed income distributions, which may attenuate gradients—particularly at the extremes. This aligns with the reduced variation observed in the right-hand panel of Figure A1 (Sect.  2). We therefore interpret the income gradient cautiously and consider it alongside an asset- and housing-based SES intended to capture longer-run material conditions. Second, in settings characterised by labour-market informality and fragmented social protection, monetary income may be an incomplete proxy for access to protective resources (e.g., schooling continuity, SRH services, and stable entitlements). Consistent with this, several demographic and household-structure features ranked above income in the individual-level classification models, suggesting that economic conditions operate alongside—and may be partly mediated by—social and demographic pathways.

From an equity perspective, these findings imply that income support alone is unlikely to yield large reductions in early motherhood unless paired with interventions that expand capabilities and reduce unequal exposure to early unions and school discontinuity. The period analysed includes major national programmes and strategies, including education-linked social protection (Progresa/Oportunidades/Prospera) [78–80], service-delivery efforts oriented to adolescent-friendly SRH [81–83], and the launch of the National Strategy for the Prevention of Adolescent Pregnancy (ENAPEA) in 2015 [84, 85]. The modest decline observed over 2000–2020 is consistent with partial gains and points to plausible “failure mechanisms” that can limit population-level impact: uneven implementation intensity across territories; limited intersectoral coordination (health–education–social protection) [86, 87]; variable service quality and persistent access barriers (including youth-friendly provision and effective contraceptive method mix) [13, 14, 16]; weak monitoring and accountability [85, 86]; and the persistence of gender norms and relationship dynamics that sustain early unions and school discontinuity [88–90]. While this study is not designed to evaluate specific programmes, the results underscore that strengthening implementation, coordination, and accountability—especially in high-risk territories—is likely important to translating policy intent into measurable equity gains [9, 85, 86]. Available success stories in this policy space tend to be local or subnational—where coordination and service delivery are stronger—highlighting the value of documenting and scaling promising territorial approaches [86, 91]. Beyond a certain material threshold, the persistence of structural and cultural factors—rather than economic hardship alone—may contribute to the continued concentration of early motherhood and intergenerational disadvantage [9, 88, 89].

Parity-specific analyses further suggest that the role of income may vary across fertility trajectories. The decline in income’s classification importance with increasing parity is consistent with the possibility that household economic resources are more strongly related to the onset of adolescent motherhood than to subsequent births, which may depend more on social norms, relationship dynamics, and post-birth access to contraception and support [92, 93]. As a result, income may capture preconditions for early motherhood more clearly than it captures later fertility dynamics among young mothers [6, 94].

At the municipal level, family and demographic structures were more strongly associated with early motherhood rates than contextual income indicators. Spatial analyses indicated persistent clustering, suggesting that place-based opportunity structures and service environments remain relevant even after adjustment for measured covariates. In the DSEM, the direct path between adult-equivalent income and early motherhood was not statistically significant, while several lagged relationships among income, schooling-related indicators, and marital-status composition were observed. These findings should be interpreted as temporal co-movements in municipal indicators rather than evidence of causal effects; nonetheless, they are policy-relevant insofar as they highlight reinforcing disadvantage in specific territorial contexts and potential leverage points for integrated interventions. These “low-opportunity traps” [5, 9, 17, 95] illustrate how economic insecurity, social norms, and limited institutional coordination can align to sustain early motherhood and reproduce inequality across generations [9, 17].

The DSEM results indicate also patterns consistent with predominantly indirect and reinforcing pathways linking income and early motherhood at the municipal level, rather than a direct contemporaneous association. Higher income at time t was associated with a lower proportion of women who were married or divorced at time t + 1, and this marital-status composition was also associated with income at time t + 1; at the same time, positive reinforcing cycles linked marital-status composition, school non-attendance, and family composition. Together, these patterns are consistent with income operating through social structures—particularly education, union formation, and household arrangements—rather than as an isolated economic factor. The absence of a statistically significant direct path between income and early motherhood does not imply independence; instead, it is consistent with a mediated and potentially equilibrium-like configuration in which these indicators co-evolve within a relatively stable social system, alongside homeostatic dynamics observed in the DSEM.

Several limitations should be recognised. First, although household income was estimated carefully using Quantile Random Forest models trained with nationally representative household surveys, these values are still estimates and not direct observations. Some measurement error may remain, which could weaken the associations, and the imputation may not fully reflect how income is shared within households, informal transfers, or short-term economic shocks. Second, the 10-year gap between census rounds limits the ability of the study to detect short-term changes or to analyse shocks and policy changes that occurred between censuses. In addition, comparable long-form census variables are not consistently available for intercensal counts, which limits the possibility of adding more time points. Third, individual and household variables are measured at the same time. Because of this, the associations observed at the individual level cannot show whether factors such as schooling, marital status, or other characteristics happened before or after early childbearing, and therefore they should not be interpreted as causal. Fourth, the DSEM is based on only three census waves. Although simple models can be estimated with three time points, this limits the ability to detect short-term dynamics and supports a descriptive, rather than causal, interpretation of the lagged parameters [51, 74]. Fifth, the “moderate” unexplained variance in the DSEM is consistent with relevant municipal determinants not captured in census-based covariates, including heterogeneity in SRH service availability and quality (e.g., youth-friendly services and contraceptive availability including LARCs), education-system conditions (school supply/quality, distance and safety), local labour-market opportunities and informality, gender norms and community-level expectations around early unions and motherhood, and exposure to violence and insecurity (including intimate partner and sexual violence). Differences in local implementation intensity of social programmes and SRH outreach may also contribute to residual variation [80, 82, 86, 87, 90]. In particular, municipalities located near expanding urban and metropolitan areas may have experienced broader socio-spatial change over time that is not fully captured by administrative continuity or by the census-based urbanicity measure alone. These limitations motivate a research agenda focused on regional heterogeneity. Furthermore, given the considerable unexplained municipal-level variance remaining after accounting for observed covariates, future work could link municipal outcomes to administrative and geospatial indicators of the SRH service environment (facility density, staffing, method availability, travel time) and use small-area estimation or hierarchical models to derive subnational estimates of SRH behaviours and social norms (e.g., contraceptive knowledge/use, unmet need, early unions) by integrating national surveys such as ENSANUT/ENADID with census covariates. Such approaches would help explain persistent spatial clusters and support the identification of scalable subnational “success stories”.

Sixth, the study uses birth and household information reported in the census, which may include reporting errors, especially among very young mothers. Seventh, although the dynamic structural models include feedback mechanisms, they cannot capture all possible confounding factors or unexpected events at the municipal level. Despite these limitations, the use of several complementary methods and a large population-based dataset helps provide useful insights into how income, inequality, and early motherhood are connected.

The evidence from Mexico provides lessons for other LMICs characterised by high inequality and fragmented welfare systems. Policy approaches that focus solely on poverty reduction or conditional cash transfers may be insufficient to disrupt the interconnected mechanisms that sustain early childbearing [96–99]. Mexico’s experience suggests that national strategies and social programmes may deliver limited aggregate change when implementation is uneven, coordination is fragmented, or service quality and access barriers persist [85]. More effective approaches must integrate educational retention, gender empowerment, and reproductive health as interdependent dimensions of social policy [100–103]. Strengthening institutional coordination across sectors is crucial, as segmented systems that tie access to health, education, and social protection to parental employment or marital status can perpetuate structural exclusion [104–106]. Territorial policies are also needed to address the localised clustering of early childbearing, combining social protection with community-based interventions that challenge gender norms and improve access to reproductive health services [5, 9, 18, 38].

In summary, early motherhood in Mexico declined only modestly between 2000 and 2020 and remained socially and territorially patterned. Income was associated with early motherhood (ages 12–24) in a non-linear way and appeared to operate alongside—rather than independently of—education, household structure, and broader institutional conditions. Equity-oriented strategies are likely to be most effective when they integrate school retention, prevention of early unions, and youth-friendly SRH services, while strengthening implementation capacity and intersectoral coordination in high-risk territories. For settings characterised by high inequality and fragmented welfare systems, Mexico’s experience suggests that sustained reductions in early childbearing will likely depend on reducing place-based gaps in opportunities and service access, rather than relying on income gains alone.

## Supplementary Information

Below is the link to the electronic supplementary material.

Supplementary Material 1: Section 1. Microdata and variable construction. Section 2. Income imputation and socioeconomic status (SES) index construction. Section 3. Results of the machine learning model. Section 4. Spatial analysis. Section 5. Additional results of DSEM model. Section 6. Sensitivity analysis

## Data Availability

The data analysed were obtained from the public repository hosted by the National Institute of Statistics and Geography of Mexico (INEGI, for its acronym in Spanish) (available at https://www.inegi.org.mx/).

## Abbreviations

LMICs: Low- and middle-income countries
LAC: Latin America and the Caribbean
SDGs: Sustainable Development Goals
ML: Machine learning
BSM: Bayesian spatial models
DSEM: Dynamic structural equation modelling
ENIGH: National Household Income and Expenditure Survey
PPP: Purchasing power parity
QRF: Quantile Random Forest
MAE: Mean absolute error
SES: Socioeconomic status
MCA: Multiple Correspondence Analysis
AUC: Area Under the Receiver Operating Characteristic Curve
LISA: Local Indicators of Spatial Association
INLA: Integrated Nested Laplace Approximation
BYM2: The Besag–York–Mollié 2
RW1: First-order random walk
DIC: Deviance Information Criterion
WAIC: Watanabe–Akaike Information Criterion
MCMC: Markov Chain Monte Carlo
95% CI: 95% confidence interval
