# Supplementary material for: Heterogeneous income gradients in early motherhood in Mexico: a population-based multi-method study, 2000–2020
Source: Int J Equity Health. 2026 Jun 25;25:216. doi: 10.1186/s12939-026-02908-w (PMC13587288; doi:10.1186/s12939-026-02908-w)
Supplement: Supplementary file 1 [file 12939_2026_2908_MOESM1_ESM.docx]

**Supplementary material**

**Section 1.** Microdata and variable construction

In Table 1, we show the definition of the variables used, as well as their construction based on the expanded samples from the censuses for the years 2000, 2010, and 2020.

Table A1. **Definition and operationalization of the variables considered in the study at the individual level**

| **Variable** | **Definition** | **Operationalisation** |
| --- | --- | --- |
| *Women's data* |  |  |
| Age of women | Years of age reported at the time of the census | Variable without additional calculation |
| Main activity | Main activity to which you dedicate most of your time, including; a) studying, b) employed, c) unpaid domestic work, d) unable to work, e) looking for work, f) retired or pensioner, g) Unemployed. | Variable without additional calculation |
| Marital status | Marital status of the woman in one of the following three categories: a) married or in a common-law union, b) separated, divorced or widowed, and c) single | Grouped variable to obtain the 3 categories described |
| Schooling lag | Years lost in schooling based on the years of schooling that should have been received according to age | This represents the estimated years of schooling lag and was constructed using conditional rules that consider the highest level of schooling attained, current age, and school attendance status. For individuals without formal education, the lag is estimated to be between 6 and 12 years, depending on their age group. Those with primary or secondary education may have between 0 and 9 years of lag, depending on whether they are currently attending school and their specific age. Individuals with upper secondary or higher education are considered up-to-date in their studies, so their lag is zero. This approach allows for the quantification of accumulated educational lag in a differentiated manner for each educational and demographic profile. |
| Health-insurance status | Report of having health insurance at the time of the census in three categories: a) with some health insurance (public or private), b) mixed (with more than one public or private health insurance), and c) without health insurance | Variable without additional calculation |
| Self-reported indigenous language | Report of speaking any language considered indigenous | Variable without additional calculation |
| Illiteracy | Report of not knowing how to read and write at the time of the census | Variable without additional calculation |
| *Household level* |  |  |
| Household head |  |  |
| Age | Age of the person reported as household head | The age of the member of each household is taken with the label assigned for household head of the kinship variable |
| Sex | Sex of the person reported as household head | The sex of the member of each household is taken with the label assigned for household head of the kinship variable |
| Schooling level | Highest schooling level of the person reported as household head | The schooling level of each household member is taken with the label assigned for household head of the kinship variable |
| Main activity | Main activity reported by the household head among the following options: a) studying, b) employed, c) unpaid domestic work, d) unable to work, e) looking for work, f) retired or pensioner, g) unemployed | The reported activity of each household member is taken with the label assigned for household head of the kinship variable |
| Household type | Type of family composition in the household in the following categories: a) unipersonal (family composed of a single person), b) nuclear family, b) extended family (by a nuclear household and one or more other relatives), e) compound family (household that includes people not related to the household head), f) corresponding family (type of family household in which at least one member has kinship ties with the household head) | Variable without additional calculation |
| Demographic dependency ratio | Total number of people in the household divided by the number of people with paid work | Number resulting from dividing the number of people in the household who do not report paid work or employment by the number of people who report paid work within the household |
| Housing type | Tenancy status of the inhabited dwelling in the following categories: a) rented, b) owned, c) other condition | Variable without additional calculation |
| Indigenous household indicator | Indigenous status of the household if any of the following conditions were met: a) the household head reported speaking an indigenous language, b) the partner of the household head reported speaking an indigenous language, or c) the father or mother of the household head reported speaking an indigenous language | A score of 1 is assigned to all households that reported any of the following conditions among their members: a) the person in the household head category reports speaking a language considered indigenous, b) the person in the wife, husband, or partner category of the household head reports speaking a language considered indigenous, or c) the father or mother of the household head reports speaking a language considered indigenous |
| Socioeconomic status (SES) | Index constructed from durable inputs and household characteristics (e.g., flooring material) using Multiple Correspondence Analysis | This variable was constructed from durable goods and household characteristics using multiple correspondence analysis. The data from 2000 was used as the base year, and the weights of each variable were used to calculate the SES for subsequent years. Further details can be found in the Income Input and SES construction section |
| Highest schooling level in household | Highest level of education attained by any member of the household | Variable without additional calculation |
| Income per adult equivalents | Imputed income per adult equivalent. Income includes earned income or any other type of income, such as social programs, remittances, rents, investments, etc | Imputed income using the random forest model, divided by the adult equivalents in the household. To calculate the adult equivalents, the household sum was calculated for the following equivalencies based on the characteristics of the household members: a) 0.67 for the household head, b) 0.33 for any other member aged 18 or older who is not the household head, c) 0.33 for members aged 14 to 18, and d) 0.2 for any member aged 13 or younger |
| Health insurance status of the household head | If the household head reports having some type of medical insurance in the following categories; a) with some health insurance (public or private), b) mixed (with more than one public or private health insurance), and c) without health insurance | The health insurance status of the person with the label assigned to the household head of the kinship variable is taken |
| Locality population size | Size of the locality where the household is located in the following categories: a) less than 2,500 inhabitants, b) from 2,501 to 10,000 inhabitants, c) from 10,000 to 99,999 inhabitants, and d) equal to or greater than 100,000 inhabitants | Variable without additional calculation |
| State identifier | State of residence identifier for the 32 states that make up the country | Variable without additional calculation |

In Table A2, we show the definition of the variables used, as well as their construction based on the expanded samples from the censuses for the years 2000, 2010, and 2020 at the municipality level.

Table A2. **Definition and operationalization of the variables considered in the study at the municipality level**

| **Variable** | **Definition** | **Operationalisation** |
| --- | --- | --- |
| Municipality identifier | Unique numeric code identifying each municipality in Mexico | Generated by concatenating the state code (Key_State × 1000) with the municipality code (Key_municipality) |
| Year | Year of observation (2000, 2010, 2020) | Variable rescaled to the [0, 1] range for model estimation |
| Cases of early motherhood | Weighted number of women (12–24 years) who reported having been pregnant at least once | Computed as the weighted sum of individuals with “with pregnancy” |
| Average income per adult equivalents | Mean imputed household income per adult equivalent | Weighted mean of individual-level, rescaled to [0, 1] |
| Proportion of married/divorced women | Share of women (12–24 years) who are married, in a free union, separated, divorced, or widowed | Weighted proportion for the specified marital status categories |
| Proportion of overcrowded households | Share of households where overcrowding was reported | Weighted proportion of individuals in households classified as overcrowded |
| Proportion of extended or composite families | Share of women living in extended or composite households | Weighted proportion using or Type housing equal to “Extended family” or “Composite family” |
| Average age of women | Average age of women (12–24 years) in the municipality | Weighted mean of age using FACTOR, rescaled to [0, 1] |
| Mean household equivalence factor | Average equivalence factor used to standardize household size to adult equivalents | Weighted mean of equivalence, rescaled to [0, 1] |
| Mean demographic dependency ratio | Average ratio of total household members to working household members | Weighted mean of dependency (household members / employed members), rescaled to [0, 1] |
| Mean age of household head | Average age of the household head across all sampled households | Weighted mean of age of household head, rescaled to [0, 1]. |
| Proportion of women not attending school | Percentage of women (12–24 years old) engaged in any activity other than studying | Weighted proportion of Activity condition in “Domestic work”, “Pensioner or retiree", "with work", "without work" |
| Mean schooling lag | Average years of schooling lag among women aged 12–24. | Weighted mean of individual schooling lag, rescaled to [0, 1] |
| Mean socioeconomic status index | Average socioeconomic status index of households | Weighted mean of SES (constructed via Multiple Correspondence Analysis) |

Section 2. **Income imputation and socioeconomic status (SES) index construction**

*Income imputation*

Although censuses measure household labor income, they omit other important income sources (remittances, social programs, family assistance) and tend to underreport income. To estimate total per capita income, we developed an imputation methodology using household, individual, and contextual variables from each census year. The imputation model was trained with the Household Income and Expenditure Survey (ENIGH) from 2000, 2010, and 2020, which provide nationally and state-representative data. ENIGH microdata from INEGI were integrated at the household level, combining individual, household, and income-expenditure databases.

We also incorporated municipal-level variables from CONEVAL and CONAPO (e.g., employment rate, access to durable goods, social lag index, health insurance coverage, Gini coefficient) and adjusted all values for inflation using the 2000 CPI as baseline. From these data, we derived indicators such as adult equivalence ratio, dependency ratio, and overcrowding. Predictors were selected based on variables consistently available in both ENIGH and censuses for all years, covering housing characteristics, durable goods, services, tenure, schooling, household composition, and local economic indicators (e.g., GDP per capita, employment structure, inequality).

We evaluated several algorithms—linear and quantile regressions, XGBoost, random forest, and quantile random forest (QRF)—selecting QRF for its superior performance in predicting per capita income. The model used 70% of data for training and 30% for testing, applying expansion factors in both phases. Validation compared predicted and observed income distributions from ENIGH and municipal-level correlations with CONEVAL’s income and multidimensional poverty indicators, showing strong concordance. In Figure A1, we show the per capita income distribution reported by ENIGN in the training sample (70%) and the per capita income predicted by the RFQ model.

Figure A1. **Comparison between income distribution reported by ENIGH vs. predicted by the RFQ models**

**Note:** Left: Per capita income (log) distribution reported by ENIGH (blue) vs. per capita income predicted by the QRF on ENIGH data (orange). Right: per capita income (log) reported by ENIGH (blue) vs. per capita income predicted by the QRF on census data (orange).

To improve accuracy, we analyzed Mean Absolute Error (MAE) by income decile across quantiles. For lower-income deciles (1–3), predictions with τ=0.3 reduced overestimation; for the top decile, τ=0.7 minimized underestimation. Final imputations used τ=0.5 globally, adjusting deciles 1–3 and 10 with τ=0.3 and τ=0.7, respectively. Advantages of QRF-based imputation include: a) capturing uncertainty by providing prediction intervals (q10–q90), b) robustness to outliers given the skewed nature of income, c) Reduced bias across income extremes through decile-specific correction.

*Socioeconomic status (SES) construction*

To construct a household-level SES index, we applied Multiple Correspondence Analysis (MCA) to categorical variables related to asset ownership, housing quality, and services. MCA reduces multidimensional data into one principal dimension that orders households along a socioeconomic gradient. Using 2000 census data as baseline, we extracted and applied the first-dimension loadings to 2010 and 2020 to ensure comparability. Selected variables included access to electricity, type of floor and dwelling, water and sanitation, overcrowding, ownership of durable goods (radio, TV, refrigerator, washing machine, car, computer), and garbage disposal method. Sampling weights were incorporated directly into the model. The first MCA dimension explained 20.5% of total variance and served as the SES indicator. Figures 2 illustrate the variance explained by dimensions and the correspondence map of variable categories for the first two dimensions.

Figure A2. **Variance explained by dimension – SES construction via MACA**

Section 3. **Results of the machine learning model**

Figure A3 shows the dependency graph that displays the predictive relationship found by the RF model between the probability of being categorized as a woman with early motherhood and marital status.

Figure A3. **Probability of early motherhood based on marital status**

Figure A4 shows the dependency graph that displays the predictive relationship found by the RF model between the probability of being categorized as a woman with early motherhood and the dependency ratio.

Figure A4. **Probability of early motherhood based on demographic dependency ratio**

Figure A5 shows the dependency graph that illustrates the predictive relationship found by the RF model between the probability of being categorized as a woman with early motherhood and the household type.

Figure A5. **Probability of early motherhood based on of household type**

Figure A6 shows the dependency graph that illustrates the predictive relationship found by the RF model between the probability of being categorized as a woman with early motherhood and main activity.

Figure A6. **Probability of early motherhood based on main activity**

Figure A7 shows the dependency graph that illustrates the predictive relationship found by the RF model between the probability of being categorized as a woman with early motherhood and the household head age.

Figure A7. **Probability of early motherhood based on household head age**

Figure A8 shows the dependency graph that illustrates the predictive relationship found by the RF model between the probability of being categorized as a woman with early motherhood and schooling lag.

Figure A8. **Probability of early motherhood based on schooling lag**

Figure A9 shows the dependency graph that illustrates the predictive relationship found by the RF model between the probability of being categorized as a woman with early motherhood and the SES.

Figure A9. **Probability of early motherhood based on SES**

**Note:** SES: Socioeconomic status.

Figure A10. **Predictors of 3+-children early motherhood by importance (random forest model)**

**Note:** The bars show the average loss of AUC when permuting each variable (B=50 permutations) with 95% confidence intervals (CI). AE: Number of adult equivalents, AUC: Area under the curve).

Figure A11. **Predictors of 2-children early motherhood by importance (random forest model)**

**Note:** The bars show the average loss of AUC when permuting each variable (B=50 permutations) with 95% confidence intervals (CI). AE: Number of adult equivalents, AUC: Area under the curve).

Figure A12. **Predictors of 1-child early motherhood by importance (random forest model)**

**Note:** The bars show the average loss of AUC when permuting each variable (B=50 permutations) with 95% confidence intervals (CI). AE: Number of adult equivalents, AUC: Area under the curve).

Figure A13. **Predictors of early motherhood by importance (random forest model) and partial dependency graph for income per AE- by age ranges**

**Panel A** 12-15 years

**Panel B** 15-19 years

**Panel C** 20-24 years

**Note:** The graph shows, in panels, the importance of the variables in the partial dependence graph of income per adult equivalent for three age groups. Panel A shows the group under 15 years, panel B shows adolescents between 15 and 19 years, and panel C shows adolescents over 20 years. As can be seen, income is much more important for adolescents over 15 years than for those under 15. AE: Number of adult equivalents, AUC: Area under the curve.

Section 4. **Spatial analysis**

Table A3. **Spatial effects distribution by Mexican state (INLA model)**

| **State** | **N Municipalities** | **Mean** | **Median** | **SD** | **Minimum** | **Maximum** | **Q1** | **Q3** | **IQR** |
| --- | --- | --- | --- | --- | --- | --- | --- | --- | --- |
| SONORA | 72.00 | 0.09 | 0.08 | 0.06 | -0.05 | 0.27 | 0.05 | 0.12 | 0.07 |
| CHIHUAHUA | 65.00 | 0.07 | 0.09 | 0.05 | -0.09 | 0.18 | 0.04 | 0.11 | 0.06 |
| COAHUILA | 38.00 | 0.06 | 0.06 | 0.03 | -0.01 | 0.13 | 0.04 | 0.08 | 0.04 |
| HIDALGO | 84.00 | 0.06 | 0.06 | 0.03 | -0.04 | 0.12 | 0.03 | 0.08 | 0.04 |
| TLAXCALA | 59.00 | 0.04 | 0.04 | 0.05 | -0.12 | 0.12 | 0.02 | 0.08 | 0.06 |
| MEXICO | 122.00 | 0.04 | 0.04 | 0.04 | -0.10 | 0.12 | 0.02 | 0.07 | 0.05 |
| BAJA CALIFORNIA | 5.00 | 0.03 | 0.04 | 0.03 | -0.01 | 0.05 | 0.02 | 0.04 | 0.03 |
| NAYARIT | 19.00 | 0.02 | 0.02 | 0.02 | -0.01 | 0.07 | 0.01 | 0.04 | 0.03 |
| QUERETARO | 18.00 | 0.02 | 0.02 | 0.03 | -0.05 | 0.07 | 0.00 | 0.05 | 0.05 |
| PUEBLA | 188.00 | 0.02 | 0.03 | 0.07 | -0.38 | 0.15 | -0.01 | 0.07 | 0.08 |
| DURANGO | 39.00 | 0.02 | 0.02 | 0.03 | -0.06 | 0.10 | 0.00 | 0.04 | 0.04 |
| COLIMA | 10.00 | 0.02 | 0.02 | 0.02 | -0.02 | 0.06 | 0.01 | 0.03 | 0.02 |
| OAXACA | 327.00 | 0.01 | 0.02 | 0.08 | -0.46 | 0.23 | -0.04 | 0.07 | 0.10 |
| BAJA CALIFORNIA SUR | 5.00 | 0.01 | 0.02 | 0.03 | -0.03 | 0.04 | -0.01 | 0.03 | 0.04 |
| MORELOS | 33.00 | 0.01 | 0.01 | 0.03 | -0.07 | 0.06 | 0.00 | 0.03 | 0.03 |
| SAN LUIS POTOSI | 57.00 | 0.01 | 0.01 | 0.04 | -0.09 | 0.09 | -0.02 | 0.04 | 0.06 |
| VERACRUZ | 203.00 | 0.00 | 0.00 | 0.04 | -0.13 | 0.11 | -0.02 | 0.03 | 0.05 |
| AGUASCALIENTES | 11.00 | 0.00 | -0.01 | 0.02 | -0.04 | 0.03 | -0.02 | 0.02 | 0.03 |
| SINALOA | 18.00 | -0.01 | 0.00 | 0.03 | -0.06 | 0.03 | -0.03 | 0.02 | 0.05 |
| CIUDAD DE MEXICO | 16.00 | -0.01 | 0.01 | 0.04 | -0.13 | 0.02 | -0.02 | 0.02 | 0.04 |
| NUEVO LEON | 50.00 | -0.01 | -0.01 | 0.04 | -0.10 | 0.13 | -0.03 | 0.00 | 0.03 |
| JALISCO | 123.00 | -0.02 | -0.01 | 0.05 | -0.16 | 0.11 | -0.05 | 0.02 | 0.07 |
| TAMAULIPAS | 41.00 | -0.03 | -0.02 | 0.03 | -0.10 | 0.03 | -0.04 | 0.00 | 0.04 |
| ZACATECAS | 55.00 | -0.03 | -0.03 | 0.03 | -0.10 | 0.07 | -0.06 | -0.01 | 0.04 |
| CHIAPAS | 110.00 | -0.03 | -0.03 | 0.04 | -0.23 | 0.10 | -0.05 | -0.01 | 0.04 |
| TABASCO | 17.00 | -0.04 | -0.04 | 0.02 | -0.09 | 0.00 | -0.05 | -0.03 | 0.03 |
| GUANAJUATO | 45.00 | -0.05 | -0.04 | 0.05 | -0.15 | 0.06 | -0.09 | -0.01 | 0.08 |
| CAMPECHE | 11.00 | -0.06 | -0.06 | 0.02 | -0.08 | -0.02 | -0.07 | -0.04 | 0.02 |
| MICHOACAN | 112.00 | -0.08 | -0.07 | 0.06 | -0.22 | 0.05 | -0.12 | -0.03 | 0.08 |
| GUERRERO | 75.00 | -0.09 | -0.08 | 0.08 | -0.66 | 0.00 | -0.10 | -0.06 | 0.04 |
| YUCATAN | 64.00 | -0.09 | -0.09 | 0.05 | -0.30 | 0.02 | -0.12 | -0.06 | 0.06 |
| QUINTANA ROO | 8.00 | -0.11 | -0.11 | 0.06 | -0.23 | -0.02 | -0.14 | -0.08 | 0.06 |

**Note:** Spatial effects represent municipality-level structured random effects from a Bayesian BYM model estimated with INLA. Positive values indicate higher-than-expected early motherhood rates after controlling for socioeconomic covariates, while negative values indicate lower-than-expected rates. States are sorted by mean spatial effect in descending order.

Section 5. **Additional results of DSEM model**

Table A4. **Correlation matrix of residual covariances (best-fitting DSEM model)**

| **Variable** | **Adolescent motherhood** | **Married/divorced** | **Compositive or extended families** | **Age of household head** | **Income per AE** | **Non-attendance at school** | **Schooling lag** |
| --- | --- | --- | --- | --- | --- | --- | --- |
| Early motherhood | 0.88 | 0.75 | 0.12 | -0.37 | -0.02 | 0.20 | 0.12 |
| Married/divorced | 0.75 | 0.85 | 0.11 | -0.38 | -0.03 | 0.25 | 0.17 |
| Compositive or extended families | 0.12 | 0.11 | 0.88 | 0.36 | 0.01 | -0.16 | -0.15 |
| Household head age | -0.37 | -0.38 | 0.36 | 0.82 | 0.06 | -0.29 | -0.24 |
| Income per AE | -0.02 | -0.03 | 0.01 | 0.06 | 0.62 | -0.24 | -0.43 |
| Non-attendance at school | 0.20 | 0.25 | -0.16 | -0.29 | -0.24 | 0.90 | 0.69 |
| Schooling lag | 0.12 | 0.17 | -0.15 | -0.24 | -0.43 | 0.69 | 0.86 |

**Note:** Values represent standardized residual covariances among latent variables from the Dynamic Structural Equation Model (DSEM). AE: Number of adult equivalents.

Section 6. **Sensitivity analysis**

Table A5. **Sensitivity analysis of Moran indices by year, by number of neighbors, crude and adjusted for covariates**

| **Year** | **Type** | **K neighbors** | **Moran index** | **p value** |
| --- | --- | --- | --- | --- |
| 2000 | raw | 2 | 0.32 | 0.00 |
| 2000 | raw | 3 | 0.31 | 0.00 |
| 2000 | raw | 4 | 0.30 | 0.00 |
| 2000 | raw | 5 | 0.29 | 0.00 |
| 2010 | raw | 2 | 0.41 | 0.00 |
| 2010 | raw | 3 | 0.39 | 0.00 |
| 2010 | raw | 4 | 0.39 | 0.00 |
| 2010 | raw | 5 | 0.37 | 0.00 |
| 2020 | raw | 2 | 0.42 | 0.00 |
| 2020 | raw | 3 | 0.39 | 0.00 |
| 2020 | raw | 4 | 0.36 | 0.00 |
| 2020 | raw | 5 | 0.34 | 0.00 |
| 2000 | adjusted | 2 | 0.12 | 0.00 |
| 2000 | adjusted | 3 | 0.10 | 0.00 |
| 2000 | adjusted | 4 | 0.10 | 0.00 |
| 2000 | adjusted | 5 | 0.10 | 0.00 |
| 2010 | adjusted | 2 | 0.28 | 0.00 |
| 2010 | adjusted | 3 | 0.26 | 0.00 |
| 2010 | adjusted | 4 | 0.25 | 0.00 |
| 2010 | adjusted | 5 | 0.25 | 0.00 |
| 2020 | adjusted | 2 | 0.24 | 0.00 |
| 2020 | adjusted | 3 | 0.24 | 0.00 |
| 2020 | adjusted | 4 | 0.22 | 0.00 |
| 2020 | adjusted | 5 | 0.22 | 0.00 |

For the sensitivity analysis of the best-fitting DSEM model, we varied the initialization of the significant parameters toward the dependent variable of blended or extended families and teenage motherhood to initializations of 0.2, 0.4, and 0.6. Simultaneously, we varied the initialization of the average age of household head toward early motherhood to -0.6, -0.4, and -0.2. The combinations resulted in a total of 9 estimated models. Figure A5 shows the 10 betas that exhibited the greatest difference across the 9 models, along with the average difference for each parameter, the standard deviation of the difference, and the maximum difference found.

Table A6. **Sensitivity analysis of the DSEM model, betas with the greatest differences out of the 9 estimated models**

| **Betas** | **Average difference** | **SD difference** | **maximum difference** |
| --- | --- | --- | --- |
| Proportion of women not attending school → Schooling lag* | 0.03 | 0.02 | 0.05 |
| Compositive or extended families → Schooling lag | 0.03 | 0.02 | 0.05 |
| Compositive or extended families (autoregressive)* | 0.02 | 0.03 | 0.04 |
| Schooling lag (autoregressive)* | 0.02 | 0.01 | 0.04 |
| Household head age → Schooling lag* | 0.02 | 0.01 | 0.04 |
| Early motherhood → Household head age | 0.01 | 0.01 | 0.02 |
| Early motherhood (autoregressive)* | 0.01 | 0.02 | 0.03 |
| Married/divorced → Early motherhood | 0.01 | 0.02 | 0.03 |
| Proportion of women not attending school → Early motherhood | 0.01 | 0.01 | 0.02 |
| Schooling lag → Proportion of women not attending school | 0.01 | 0.01 | 0.03 |

**Note:** Betas marked with * are significant betas; those without the sign are changes in betas but are not significant.
